# 3off2: A network reconstruction algorithm based on 2-point and 3-point information statistics

**DOI:** 10.1186/s12859-015-0856-x

**Published:** 2016-01-20

**Authors:** Séverine Affeldt, Louis Verny, Hervé Isambert

**Affiliations:** 1 Institut Curie, PSL Research University, CNRS, UMR168, 26 rue d’Ulm, Paris, 75005 France; 2 Sorbonne Universités, UPMC Univ Paris 06, 4, Place Jussieu, Paris, 75005 France

**Keywords:** Network reconstruction, Hybrid inference method, Information theory, Hematopoiesis

## Abstract

**Background:**

The reconstruction of reliable graphical models from observational data is important in bioinformatics and other computational fields applying network reconstruction methods to large, yet finite datasets. The main network reconstruction approaches are either based on Bayesian scores, which enable the ranking of alternative Bayesian networks, or rely on the identification of structural independencies, which correspond to missing edges in the underlying network. Bayesian inference methods typically require heuristic search strategies, such as hill-climbing algorithms, to sample the super-exponential space of possible networks. By contrast, constraint-based methods, such as the PC and IC algorithms, are expected to run in polynomial time on sparse underlying graphs, provided that a correct list of conditional independencies is available. Yet, in practice, conditional independencies need to be ascertained from the available observational data, based on adjustable statistical significance levels, and are not robust to sampling noise from finite datasets.

**Results:**

We propose a more robust approach to reconstruct graphical models from finite datasets. It combines constraint-based and Bayesian approaches to infer structural independencies based on the ranking of their most likely contributing nodes. In a nutshell, this local optimization scheme and corresponding 3off2 algorithm iteratively “take off” the most likely conditional 3-point information from the 2-point (mutual) information between each pair of nodes. Conditional independencies are thus derived by progressively collecting the most significant indirect contributions to all pairwise mutual information. The resulting network skeleton is then partially directed by orienting and propagating edge directions, based on the sign and magnitude of the conditional 3-point information of unshielded triples. The approach is shown to outperform both constraint-based and Bayesian inference methods on a range of benchmark networks. The 3off2 approach is then applied to the reconstruction of the hematopoiesis regulation network based on recent single cell expression data and is found to retrieve more experimentally ascertained regulations between transcription factors than with other available methods.

**Conclusions:**

The novel information-theoretic approach and corresponding 3off2 algorithm combine constraint-based and Bayesian inference methods to reliably reconstruct graphical models, despite inherent sampling noise in finite datasets. In particular, experimentally verified interactions as well as novel predicted regulations are established on the hematopoiesis regulatory networks based on single cell expression data.

**Electronic supplementary material:**

The online version of this article (doi:10.1186/s12859-015-0856-x) contains supplementary material, which is available to authorized users.

## Background

Two types of reconstruction method for directed networks have been developed and applied to a variety of experimental datasets. These methods are either based on Bayesian scores [1, 2] or rely on the identification of structural independencies, which correspond to missing edges in the underlying network [3, 4].

Bayesian inference approaches have the advantage of allowing for quantitative comparisons between alternative networks through their Bayesian scores but they are limited to rather small causal graphs due to the super-exponential space of possible directed graphs to sample [1, 5, 6]. Hence, Bayesian inference methods typically require either suitable prior restrictions on the structures [7, 8] or heuristic search strategies such as hill-climbing algorithms [9–11].

By contrast, structure learning algorithms based on the identification of structural constraints typically run in polynomial time on sparse underlying graphs. These so-called constraint-based approaches, such as the PC [12] and IC [13] algorithms, do not score and compare alternative networks. Instead they aim at ascertaining conditional independencies between variables to directly infer the Markov equivalent class of all causal graphs compatible with the available observational data. Yet, these methods are not robust to sampling noise in finite datasets as early errors in removing edges from the complete graph typically trigger the accumulation of compensatory errors later on in the pruning process. This cascading effect makes the constraint-based approaches sensitive to the adjustable significance level *α*, required for the conditional independence tests. In addition, traditional constraint-based methods are not robust to the order in which the conditional independence tests are processed, which prompted recent algorithmic improvements intending to achieve order-independence [14].

In this paper, we report a novel network reconstruction method, which exploits the best of these two types of structure learning approaches. It combines constraint-based and Bayesian frameworks to reliably reconstruct graphical models despite inherent sampling noise in finite observational datasets. To this end, we have developed a robust information-theoretic method to confidently ascertain structural independencies in causal graphs based on the ranking of their most likely contributing nodes. Conditional independencies are derived using an iterative search approach that identifies the most significant indirect contributions to all pairwise mutual information between variables. This local optimization algorithm, outlined below, amounts to iteratively subtracting the most likely conditional 3-point information from 2-point information between each pair of nodes. The resulting network skeleton is then partially directed by orienting and propagating edge directions, based on the sign and magnitude of the conditional 3-point information of unshielded triples. Identifying structural independencies within such a maximum likelihood framework circumvents the need for adjustable significance levels and is found to be more robust to sampling noise from finite observational data, even when compared to constraint-based methods intending to resolve the order-dependence on the variables [14].



### Constraint-based methods

Constraint-based approaches, such as the PC [12] and IC [13] algorithms, infer causal graphs from observational data, by searching for conditional independencies among variables. Under the Markov and Faithfulness assumptions, these algorithms return a Complete Partially Directed Acyclic Graph (CPDAG) that represents the Markov equivalent class of the underlying causal structure [3, 4]. They proceed in three steps detailed in Algorithm 1:

- 1) inferring unnecessary edges and associated separation sets to obtain an undirected skeleton.
- 2) orienting unshielded triples as v-structures if their middle node is not in the separation set (*R*_0_).
- 3) propagating as many orientations as possible following propagation rules (*R*_1 − 3_), which prevents the orientation of additional v-structures (*R*_3_) and directed cycles (*R*_2 − 3_) [15].

However, as previously stated, the sensitivity of the constraint-based methods to the adjustable significance level *α* used for the conditional independence tests and to the order in which the variables are processed (step 1) favors the accumulation of errors when the search procedure relies on finite observational data.

In this paper, we aim at improving constraint-based methods, Algorithm 1, by uncovering the most reliable conditional independencies supported by the (finite) available data, based on a quantitative information theoretic framework.

### Maximum likelihood methods

The maximum likelihood ${\mathcal {L_{G}}}$ is related to the cross entropy $H({\mathcal {G,D}})=-\sum _{\{x_{i}\}}p(\{x_{i}\})\log (q(\{x_{i}\}))$ between the “true” probability distribution *p*({*x*_*i*_}) from the data $\mathcal {D}$ and the approximate probability distribution $q(\{x_{i}\})=\prod _{i}p(x_{i}\vert \{Pa_{x_{i}}\})$ generated by the Bayesian network ${\mathcal {G}}$ with specific parent nodes $\{Pa_{x_{i}}\}$ for each node *x*_*i*_, leading to [16],

(1) $$\begin{array}{*{20}l} {\mathcal{L_{G}}}=e^{-N H({\mathcal{G,D}})}=e^{-N \sum_{i} H\left(x_{i}\vert \left\{{Pa}_{x_{i}}\right\}\right)}  \end{array} $$

where $\sum _{i} H(x_{i}\vert \{\!{Pa}_{x_{i}}\!\})$ is the (conditional) entropy of the underlying causal graph. This enables to score and compare alternative models through their maximum likelihood ratio as,

(2) $$\begin{array}{*{20}l} {\mathcal{L_{G'}} \over \mathcal{L_{G}}}=e^{-N \sum_{i} \left(H\left(x_{i}\vert \left\{Pa'_{x_{i}}\right\}\right)-H\left(x_{i}\vert \left\{{Pa}_{x_{i}}\right\}\right)\right)}  \end{array} $$

Note, in particular, that the significance level of the Maximum likelihood approach is set by the number *N* of independent observational data points, as detailed in the Methods Section below.

## Methods

### Information theoretic framework

#### Inferring isolated v-structures vs non-v-structures from 3-point and 2-point information

**Fig. 1:**
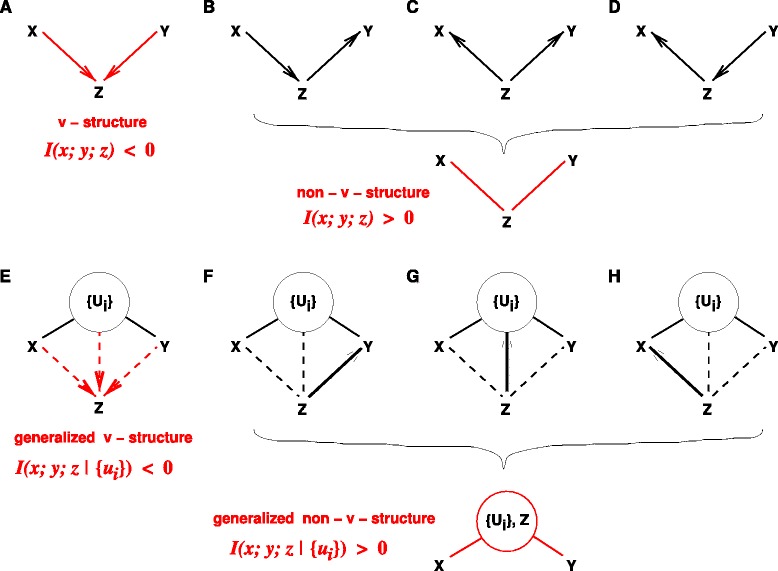
Inference of v-structures versus non-v-structures by 3-point information from observational data. **a** Isolated v-structures are predicted for *I*(*x*;*y*;*z*) < 0, and (**b**–**d**) isolated non-v-structures for *I*(*x*;*y*;*z*) > 0. **e** Generalized v-structures are predicted for *I*(*x*;*y*;*z*|{*u*_*i*_}) < 0 and (**f**–**h**) generalized non-v-structures for *I*(*x*;*y*;*z*|{*u*_*i*_}) > 0. In addition, as *I*(*x*;*y*;*z*|{*u*_*i*_}) are invariant upon *xyz* permutations, the global orientation of v-structures and non-v-structures also requires to find the most likely base of the *xyz* triple. Choosing the base *xy* with the lowest conditional mutual information, *i.e.*, *I*(*x*;*y*|{*u*_*i*_})= min*xyz*(*I*(*s*;*t*|{*u*_*i*_})), is found to be consistent with the Data Processing Inequality expected for (generalized) non-v-structures in the limit of infinite dataset, see main text. In practice, given a finite dataset, the inference of (generalized) v-structures *versus* non-v-structures can be obtained by replacing 3-point and 2-point information terms *I*(*x*;*y*|{*u*_*i*_}) and *I*(*x*;*y*;*z*|{*u*_*i*_}) by shifted equivalents, *I* ^′^(*x*;*y*|{*u*_*i*_}) and *I* ^′^(*x*;*y*;*z*|{*u*_*i*_}), including finite size corrections, see text (Eqs. 23 & 24)

Applying the previous likelihood definition, Eq. 1, to isolated v-structures (Fig. 1a) and Markov equivalent non-v-structures (Fig. 1b–d), one obtains,

(3) $$\begin{array}{*{20}l} {{\mathcal{L}_{\sf v}(xy)}} &= e^{-N\left[H(z\vert x,y)+H(x)+H(y)\right]}\\ &= e^{-N\left[H(x,y,z)+I(x;y)\right]}  \end{array} $$

where *I*(*x*;*y*)=*H*(*x*)+*H*(*y*)−*H*(*x*,*y*) is the 2-point mutual information between *x* and *y*, and,

(4) $$\begin{array}{*{20}l} {{\mathcal{L}_{\sf nv}(xy)}}&= e^{-N\left[H(x\vert z)+H(y\vert z)+H(z)\right]}\\ &= e^{-N\left[H(x,y,z)+I(x;y\vert z)\right]}  \end{array} $$

where *I*(*x*;*y*|*z*)=*H*(*x*|*z*)+*H*(*y*|*z*)−*H*(*x*,*y*|*z*) is the conditional mutual information between *x* and *y* given *z*. Hence, one obtains the likelihood ratio,

(5) $$\begin{array}{*{20}l} {{\mathcal{L}_{\sf v}}(xy)\over {\mathcal{L}_{\sf nv}}(xy)}=e^{-N \left[I(x;y)-I(x;y\vert z)\right]}=e^{-N I(x;y;z)}  \end{array} $$

where we introduced the 3-point information function, *I*(*x*;*y*;*z*)=*I*(*x*;*y*)−*I*(*x*;*y*|*z*), which is in fact invariant upon permutations between *x*,*y* and *z*, as seen in terms of entropy functions,

(6) $$\begin{array}{*{20}l} I(x;y;z)&=H(x)+H(y)+H(z)-H(x,y)\\ &\quad -H(x,z)-H(y,z)+H(x,y,z) \end{array} $$

As long recognized in the field [17, 18], 3-point information, *I*(*x*;*y*;*z*), can be positive or negative (if *I*(*x*;*y*)<*I*(*x*;*y*|*z*)), unlike 2-point mutual information, which are always positive, $I(x;y)\geqslant 0$.

More precisely, Eq. 5 demonstrates that the sign and magnitude of 3-point information provide a quantitative estimate of the relative likelihoods of isolated v-structures versus non-v-structures, which are in fact independent of their actual non-connected bases *xy*, *xz* or *yz*,

(7) $$\begin{array}{*{20}l} {{\mathcal{L}_{\sf v}}(xy)\over {\mathcal{L}_{\sf nv}}(xy)}={{\mathcal{L}_{\sf v}}(xz)\over {\mathcal{L}_{\sf nv}}(xz)}={{\mathcal{L}_{\sf v}}(yz)\over {\mathcal{L}_{\sf nv}}(yz)}=e^{-N I(x;y;z)}  \end{array} $$

Hence, a significantly negative 3-point information, *I*(*x*;*y*;*z*)<0, implies that a v-structure is more likely than a non-v-structure given the observed correlation data. Conversely, a significantly positive 3-point information, *I*(*x*;*y*;*z*)>0, implies that a non-v-structure model is more likely than a v-structure model.

Yet, as noted above, 3-point information, *I*(*x*;*y*;*z*), being symmetric by construction, it cannot indicate how to orient v-structures or non-v-structures over the *xyz* triple. To this end, it is however straightforward to show that the most likely base (*xy*, *xz* or *yz*) of the local v-structure or non-v-structure corresponds to the pair with lowest mutual information, *e.g.*, $I(x;y)=\min _{\textit {xyz}}\bigl (I(s;t)\bigr)$, as shown by the likelihood ratios,

(8) $$\begin{array}{*{20}l} {{\mathcal{L}_{\sf v}}(xy)\over {\mathcal{L}_{\sf v}}(st)}={{\mathcal{L}_{\sf nv}}(xy)\over {\mathcal{L}_{\sf nv}}(st)}={e^{-N I(x;y)} \over e^{-N I(s;t)}} \end{array} $$

Note, in particular, that choosing the base with the lowest mutual information is consistent with the Data Processing Inequality expected for non-v-structures, Fig. 1b–d.

Hence, combining 3-point and 2-point information allows to determine the likelihood and the base of isolated v-structures *versus* non-v-structures. But how to extend such simple results to identify local v-structures and non-v-structures embedded within an entire graph ${\mathcal {G}}$?

#### Inferring embedded v-structures vs non-v-structures from conditional 3-point and 2-point information

To go from isolated to embedded v-structures and non-v-structures within a DAG ${\mathcal {G}}$, we will consider the Markov equivalent CPDAG of ${\mathcal {G}}$ and introduce generalized v-structures and non-v-structures, Fig. 1e–h. We will demonstrate that their relative likelihood, given the available observational data, can be estimated from the sign and magnitude of a conditional 3-point information, *I*(*x*;*y*;*z*|{*u*_*i*_}), Eq. 11. This will extend our initial result valid for isolated v-structures and non-v-structures, Eq. 7.

Let’s consider a pair of non-neighbor nodes *x*,*y* with a set of upstream nodes {*u*_*i*_}_*n*_, where each node *u*_*i*_ has at least one direct connection to *x* (*u*_*i*_→*x*) or *y* (*u*_*i*_→*y*) or to another upstream node *u*_*j*_∈{*u*_*i*_}_*n*_ (*u*_*i*_→*u*_*j*_) or only undirected links to these nodes (*u*_*i*_−*x*, *u*_*i*_−*y* or *u*_*i*_−*u*_*j*_). Thus, given *x*,*y* and a set of upstream nodes {*u*_*i*_}_*n*_, any additional node *z* can either be:

- *i)* at the apex of a generalized v-structure, if *all* existing connections between *x*, *y*, {*u*_*i*_}_*n*_ and *z* are directed and point towards *z*, Fig. 1e, or else,
- *ii)z* has *at least one* undirected link with *x*, *y* or one of the upstream nodes *u*_*i*_ (*z*−*x*, *z*−*y* or *z*−*u*_*i*_) *or at least one* directed link pointing towards these nodes (*z*→*x*, *z*→*y* or *z*→*u*_*i*_), Fig. 1f–h. In such a case, *z* might contribute to the mutual information *I*(*x*;*y*) and should be included in the set of upstream nodes {*u*_*i*_}_*n*_, thereby defining a generalized non-v-structure, Figs. 1f–h.

Then, similarly to the case of an isolated v-structure (Eq. 3), the maximum likelihood ${\mathcal {L}_{\sf v}(xy)}$ of a generalized v-structure pointing towards *z* from a base *xy* with upstream nodes {*u*_*i*_}_*n*_ can be expressed as,

(9) $$\begin{array}{*{20}l} {{\mathcal{L}_{\sf v}(xy)}}&= e^{-N \left[H(z\vert x,y,\{u_{i}\})+H(\!x\vert\{u_{i}\})+H(y\vert\{u_{i}\})+H(\{u_{i}\})\right]}\\ &= e^{-N \left[H(x,y,z,\{u_{i}\})+I(x;y\vert\{u_{i}\})\right]}  \end{array} $$

where *I*(*x*;*y*|{*u*_*i*_}) is the conditional mutual information between *x* and *y* given {*u*_*i*_}, *I*(*x*;*y*|{*u*_*i*_})=*H*(*x*|{*u*_*i*_})+*H*(*y*|{*u*_*i*_})−*H*(*x*,*y*|{*u*_*i*_})−*H*({*u*_*i*_}).

Likewise, the maximum likelihood ${\mathcal {L}_{\sf nv}(xy)}$ of a generalized non-v-structure of base *xy* with upstream nodes {*u*_*i*_}_*n*_ and *z* can be expressed as,

(10) $$\begin{array}{*{20}l} {{\mathcal{L}_{\sf nv}(xy)}}&= e^{-N\left[H(x\vert z,\{u_{i}\})+H(y\vert z,\{u_{i}\})+H(z,\{u_{i}\})\right]}\\ &= e^{-N\left[H(x,y,z,\{u_{i}\})+I(x;y\vert z,\{u_{i}\})\right]} \end{array} $$

where *I*(*x*;*y*|*z*,{*u*_*i*_})=*H*(*x*|*z*,{*u*_*i*_})+*H*(*y*|*z*,{*u*_*i*_})−*H*(*x*,*y*|*z*, {*u*_*i*_})−*H*(*z*,{*u*_*i*_}) is the conditional mutual information between *x* and *y* given *z* and {*u*_*i*_}. Hence,

(11) $$\begin{array}{*{20}l} {{\mathcal{L}_{\sf v}(xy)} \over {\mathcal{L}_{\sf nv}(xy)}}=e^{-N I(x;y;z\vert\{u_{i}\})}  \end{array} $$

where we introduced the conditional 3-point information, *I*(*x*;*y*;*z*|{*u*_*i*_})=*I*(*x*;*y*|{*u*_*i*_})−*I*(*x*;*y*|*z*,{*u*_*i*_}).

Hence, a significantly negative conditional 3-point information, *I*(*x*;*y*;*z*|{*u*_*i*_})<0, implies that a generalized v-structure is more likely than a generalized non-v-structure given the available observational data. Conversely, a significantly positive conditional 3-point information, *I*(*x*;*y*;*z*|{*u*_*i*_})>0, implies that a generalized non-v-structure model is more likely than a generalized v-structure model.

Yet, as the conditional 3-point information, *I*(*x*;*y*;*z*|{*u*_*i*_}), is in fact invariant upon permutations between *x*,*y* and *z*, it cannot indicate how to orient embedded v-structures or non-v-structures over the *xyz* triple, as already noted in the case of isolated v-structures and non-v-structures, above.

However, the most likely base (*xy*, *xz* or *yz*) of the embedded v-structure or non-v-structure corresponds to the least correlated pair conditioned on {*u*_*i*_}, *e.g.*, $I(x;y\vert \{u_{i}\})=\min _{\textit {xyz}}\bigl (I(s;t\vert \{u_{i}\})\bigr)$, as shown with the following likelihood ratios,

(12) $$\begin{array}{*{20}l} {{\mathcal{L}_{\sf v}}(xy)\over {\mathcal{L}_{\sf v}}(st)}={{\mathcal{L}_{\sf nv}}(xy)\over {\mathcal{L}_{\sf nv}}(st)}={e^{-N I(x;y\vert\{u_{i}\})} \over e^{-N I(s;t\vert\{u_{i}\})}}  \end{array} $$

Note, in particular, that choosing the base with the lowest conditional mutual information, *e.g.*, $I(x;y\vert \{u_{i}\})=\min _{\textit {xyz}}\bigl (I(s;t\vert \{u_{i}\})\bigr)$, is consistent with the Data Processing Inequality expected for the generalized non-v-structure of Fig. 1f–h, $I(x;y) \leqslant \min \bigl (I(x;z,\{u_{i}\}), I(z,\{u_{i}\};y)\bigr)$, as shown below for *I*(*x*;*y*) and *I*(*x*;*z*,{*u*_*i*_}), by subtracting *I*(*x*;*y*;*z*|{*u*_*i*_}) on each side of the inequality *I*(*x*;*y*|{*u*_*i*_})≤*I*(*x*;*z*|{*u*_*i*_}), leading to,

(13) $$\begin{array}{*{20}l} I(x;y\vert z,\{u_{i}\}) &\leqslant I(x;z\vert\{u_{i}\},y)\\ &\leqslant I(x;z\vert\{u_{i}\},y)+I(x;\{u_{i}\}\vert y)\\ &\leqslant I(x;z,\{u_{i}\}\vert y)\\ I(x;y) &\leqslant I(x;z,\{u_{i}\}) \end{array} $$

where we have used the chain rule, *I*(*x*;*z*,{*u*_*i*_}|*y*)=*I*(*x*;*z*|{*u*_*i*_},*y*)+*I*(*x*;{*u*_*i*_}|*y*), before adding *I*(*x*;*y*;*z*,{*u*_*i*_}) on each side of the inequality. The corresponding inequality holds between *I*(*x*;*y*) and *I*(*z*,{*u*_*i*_};*y*), implying the Data Processing Inequality.

#### Finite size corrections of maximum likelihood

Maximum likelihood ratios, such as Eq. 2, suggest that 1/*N* sets the significance level of the maximum likelihood approach, as $H({\mathcal {G,D}})-H({\mathcal {G',D}}) \gg 1/N$ should imply a significant improvement of the underlying model ${\mathcal {G'}}$ over ${\mathcal {G}}$. In practice, however, there are ${\mathcal {O}}(\log (N)/N)$ corrections coming from the proper normalization of maximum likelihoods (see Appendix),

(14) $$\begin{array}{*{20}l} {\mathcal{L_{G}}}={e^{-N \sum_{i} H\left(x_{i}\vert \left\{\text{Pa}_{x_{i}}\right\}\right)}\over Z(\mathcal{G,D})}  \end{array} $$

The model $\mathcal {G}$ can then be compared to the alternative model $\mathcal {G}_{\setminus {x\to y}}$ with one missing edge *x*→*y* using the maximum likelihood ratio,

(15) $$\begin{array}{*{20}l} {\mathcal{L}_{\mathcal{G}_{\setminus{x\to y}}}\over \mathcal{L}_{\mathcal{G}}}={e^{-N I(x;y\vert \{\text{Pa}_{y}\}_{\setminus{x}})}{Z(\mathcal{G,D})\over Z(\mathcal{G}_{\setminus{x\to y}},\mathcal{D})}}  \end{array} $$

where *I*(*x*;*y*|{Pa_*y*_}_∖*x*_)=*H*(*y*|{Pa_*y*_}_∖*x*_)−*H*(*y*|{Pa_*y*_}).

Then, following the rationale of constraint-based approaches, Eq. 15 can be reformulated by replacing the parent nodes {Pa_*y*_}_∖*x*_ with an unknown separation set {*u*_*i*_} to be learnt simultaneously with the missing edge candidate *xy*,

(16) $$\begin{array}{*{20}l} {\mathcal{L}_{\mathcal{G}_{\setminus{xy\vert \{u_{i}\}}}}\over \mathcal{L}_{\mathcal{G}}}&= {e^{-N I(x;y\vert \{u_{i}\})+ k_{x;y\vert \{u_{i}\}}}} \end{array} $$

(17) $$\begin{array}{*{20}l} k_{x;y\vert \{u_{i}\}}&= \log\big(Z(\mathcal{G,D})/Z(\mathcal{G}_{\setminus{xy\vert \{u_{i}\}}},\mathcal{D})\big) \end{array} $$

where the factor $k_{x;y\vert \{u_{i}\}}>0$ tends to limit the complexity of the models by favoring fewer edges. Namely, the condition, $I(x;y\vert \{u_{i}\})< k_{x;y\vert \phantom {\dot {i}\!}\{u_{i}\}}/N$, implies that simpler models compatible with the structural independency, *x* ⊥ ⊥ *y*|{*u*_*i*_}, are more likely than model $\mathcal {G}$, given the finite available dataset. This replaces the ‘perfect’ conditional independency condition, *I*(*x*;*y*|{*u*_*i*_})=0, valid in the limit of an infinite dataset, *N*→*∞*. A common complexity criteria in model selection is the Bayesian Information Criteria (BIC) or Minimal Description Length (MDL) criteria [19, 20],

(18) $$ k^{^{\sf MDL}}_{x;y\vert \{u_{i}\}}={1\over 2}(r_{x}-1)(r_{y}-1)\prod_{i}r_{u_{i}}\log N   $$

where *r*_*x*_,*r*_*y*_ and $r_{u_{i}}$ are the number of levels of the corresponding variables. The MDL complexity, Eq. 18, is simply related to the normalisation constant of the distribution reached in the asymptotic limit of a large dataset *N*→*∞* (Laplace approximation). However, this limit distribution is only reached for very large datasets in practice.

Alternatively, the normalisation of the maximum likelihood can also be done over all possible datasets including the same number of data points to yield a (universal) Normalized Maximum Likelihood (NML) criteria [21, 22] and its decomposable [23, 24] and *xy*-symmetric version, $k^{^{\sf NML}}_{x;y\vert \{u_{i}\}}$, defined in the Appendix.

Then, incrementing the separation set of *xy* from {*u*_*i*_} to {*u*_*i*_}+*z* leads to the following likelihood ratio,

(19) $$\begin{array}{*{20}l} {\mathcal{L}_{\mathcal{G}_{\setminus{xy}\vert \{u_{i}\},z}}\over \mathcal{L}_{\mathcal{G}_{\setminus{xy}\vert \{u_{i}\}}}}={e^{N I(x;y;z\vert \{u_{i}\})+ k_{x;y;z\vert \{u_{i}\}}}}  \end{array} $$

with *I*(*x*;*y*;*z*|{*u*_*i*_})=*I*(*x*;*y*|{*u*_*i*_})−*I*(*x*;*y*|{*u*_*i*_},*z*) and where we introduced a 3-point conditional complexity, $k_{x;y;z\vert \{u_{i}\}}$, defined similarly as the difference between the 2-point conditional complexities,

(20) $$\begin{array}{*{20}l} k_{x;y;z\vert \{u_{i}\}}=k_{x;y\vert \{u_{i}\},z}-k_{x;y\vert \{u_{i}\}}  \end{array} $$

However, unlike 3-point information, *I*(*x*;*y*;*z*|{*u*_*i*_}), 3-point complexities are always positive, $k_{x;y;z\vert \{u_{i}\}}>0$, provided that there are at least two levels for each implicated node *ℓ*∈*x*,*y*,*z*,{*u*_*i*_}, *i.e.*$r_{\ell } \geqslant 2$.

Hence, we can define the shifted 2-point and 3-point information in Eqs. 16 & 19 for finite datasets as,

(21) $$\begin{array}{*{20}l} I^{\prime}(x;y\vert \{u_{i}\})&= I(x;y\vert \{u_{i}\})-{k_{x;y\vert \{u_{i}\}}\over N} \end{array} $$

(22) $$\begin{array}{*{20}l} I^{\prime}(x;y;z\vert \{u_{i}\})&= I(x;y;z\vert \{u_{i}\})+{k_{x;y;z\vert \{u_{i}\}}\over N}  \end{array} $$

This leads to the following maximum likelihood ratios equivalent to Eqs. 11 & 12 for v-structure over non-v-structure and between alternative bases,

(23) $$\begin{array}{*{20}l} {{\mathcal{L}_{\sf v}}(xy)\over {\mathcal{L}_{\sf nv}}(xy)}&= {e^{-N I^{\prime}(x;y;z\vert \{u_{i}\})}}  \end{array} $$

(24) $$\begin{array}{*{20}l} {{\mathcal{L}_{\sf v}}(xy)\over {\mathcal{L}_{\sf v}}(st)}&= {{\mathcal{L}_{\sf nv}}(xy)\over {\mathcal{L}_{\sf nv}}(st)}={e^{-N I^{\prime}(x;y\vert \{u_{i}\})} \over e^{-N I^{\prime}(s;t\vert \{u_{i}\})}}  \end{array} $$

Hence, given a finite dataset, a significantly negative conditional 3-point information, corresponding to *I*^′^(*x*;*y*;*z*|{*u*_*i*_})<0, implies that a v-structure *x*→*z*←*y* is more likely than a non-v-structure provided that the structural independency, *x* ⊥ ⊥ *y*|{*u*_*i*_}, is also confidently established as, *I*^′^(*x*;*y*|{*u*_*i*_})<0. By contrast, a significantly positive conditional 3-point information corresponds to *I*^′^(*x*;*y*;*z*|{*u*_*i*_})>0 and implies that a non-v-structure model is more likely than a v-structure model, given the available observational data.

#### Probability estimate of indirect contributions to mutual information

The previous results enable us to estimate the probability of a node *z* to contribute to the conditional mutual information *I*(*x*;*y*|{*u*_*i*_}), by combining the probability, *P*_*nv*_(*xyz*|{*u*_*i*_}), that the triple *xyz* is a generalized non-v-structure conditioned on {*u*_*i*_} and the probability, *P*_*b*_(*xy*|{*u*_*i*_}), that its base is *xy*, where,

(25) $$\begin{array}{*{20}l} P_{\sf nv}(xyz\vert\{u_{i}\})&= {{\mathcal{L}_{\sf nv}}(xy)\over {\mathcal{L}_{\sf nv}}(xy)+{\mathcal{L}_{\sf v}}(xy)} \end{array} $$

(26) $$\begin{array}{*{20}l} P_{\sf b}(xy\vert\{u_{i}\})&= {{\mathcal{L}_{\sf nv}}(xy)\over {\mathcal{L}_{\sf nv}}(xy)+{\mathcal{L}_{\sf nv}}(xz)+{\mathcal{L}_{\sf nv}}(yz)} \end{array} $$

that is, using Eqs. 23 & 24 including finite size corrections of the maximum likelihoods,

(27) $$\begin{array}{*{20}l} P_{\sf nv}(xyz\vert\{u_{i}\})&= {1\over {\scriptsize 1+e^{-N{{I^{\prime}(x;y;z\vert\{u_{i}\})}}}}} \end{array} $$

(28) $$\begin{array}{*{20}l} P_{\sf b}(xy\vert\{u_{i}\})&= {1\over {1+ {e^{-N{{I^{\prime}(x;z\vert \{u_{i}\})}}}\over e^{-N{{I^{\prime}(x;y\vert \{u_{i}\})}}}}+{e^{-N{{I^{\prime}(y;z\vert \{u_{i}\})}}}\over e^{-N{{I^{\prime}(x;y\vert \{u_{i}\})}}}}}} \end{array} $$

Then, various alternatives to combine *P*_*nv*_(*xyz*|{*u*_*i*_}) and *P*_*b*_(*xy*|{*u*_*i*_}) exist to estimate the overall probability that the additional node *z* indirectly contributes to *I*(*x*;*y*|{*u*_*i*_}). One possibility is to choose the lower bound *S*_*lb*_(*z*;*xy*|{*u*_*i*_}) of *P*_*nv*_(*xyz*|{*u*_*i*_}) and *P*_*b*_(*xy*|{*u*_*i*_}), since both conditions need to be fulfilled to warrant that *z* indeed contributes to *I*(*x*;*y*|{*u*_*i*_}),

(29) $$\begin{array}{*{20}l} S_{\sf lb}(z;xy\vert\{u_{i}\})=\min\big[P_{\sf nv}(xyz\vert\{u_{i}\}), P_{\sf b}(xy\vert \{u_{i}\})\big]\;\;\;&&\end{array} $$

The pair of nodes *xy* with the most likely contribution from a third node *z* can then be ordered according to their rank *R*(*xy*;*z*|{*u*_*i*_}) defined as,

(30) $$\begin{array}{*{20}l} R(xy;z\vert\{u_{i}\})=\max_{z}\big(S_{\sf lb}(z;xy\vert\{u_{i}\})\big)  \end{array} $$

and *z* can be iteratively added to the set of contributing nodes (*i.e.* {*u*_*i*_}←{*u*_*i*_}+*z*) of the top link *xy*=argmax_*xy*_*R*(*xy*;*z*|{*u*_*i*_}) to progressively recover the most significant indirect contributions to all pairwise mutual information in a causal graph, as outlined below.

#### Robust inference of conditional independencies using the 3off2 scheme

The previous results can be used to provide a robust inference method to identify conditional independencies and, hence, reconstruct the skeleton of underlying causal graphs from finite available observational data. The approach follows the spirit of constraint-based methods, such as the PC or IC algorithms, but recovers conditional independencies following an evolving ranking of the network edges, *R*(*xy*;*z*|{*u*_*i*_}), defined in Eq. 30.

All in all, this amounts to perform a generic decomposition for each mutual information term, *I*(*x*;*y*), by introducing a succession of node candidates, *u*_1_,*u*_2_,…, *u*_*n*_, that are likely to contribute to the overall mutual information between the pair *x* and *y*, as,

(31) $$\begin{array}{*{20}l} I(x;y)&= I(x;y; u_{1})+I(x;y\vert u_{1})\\ &= I(x;y; u_{1})+I(x;y;u_{2}\vert u_{1})+\ldots\\ &\quad \ldots+I(x;y;u_{n}\vert\{u_{i}\}_{n-1})+I(x;y\vert\{u_{i}\}_{n}) \end{array} $$

or equivalently between the shifted 2-point and 3-point information terms including finite size corrections (Eq. 22),

(32) $$\begin{array}{*{20}l} I^{\prime}(x;y)&= I^{\prime}(x;y; u_{1})+I^{\prime}(x;y;u_{2}\vert u_{1})+\ldots\\ &\quad +I^{\prime}(x;y;u_{n}\vert\{u_{i}\}_{n-1})+I^{\prime}(x;y\vert\{u_{i}\}_{n}) \end{array} $$

Hence, given a significant mutual information between *x* and *y*, *I*^′^(*x*;*y*)>0, we will search for possible structural independencies, *i.e.I*^′^(*x*;*y*|{*u*_*i*_}_*n*_)<0, by iteratively “*taking off*” conditional 3-point information terms from the initial 2-point (mutual) information, *I*^′^(*x*;*y*), as

(33) $$\begin{array}{*{20}l} I^{\prime}(x;y\vert\{u_{i}\}_{n})&= I^{\prime}(x;y)-I^{\prime}(x;y; u_{1})-I^{\prime}(x;y; u_{2}\vert u_{1})\\ &\quad -\ldots-I^{\prime}(x;y;u_{n}\vert\{u_{i}\}_{n-1})  \end{array} $$

and similarly with non-shifted 2-point and 3-point information,

(34) $$\begin{array}{*{20}l} I(x;y\vert\{u_{i}\}_{n})&= I(x;y)-I(x;y; u_{1})-I(x;y; u_{2}\vert u_{1})\\ &\quad -\ldots-I(x;y;u_{n}\vert\{u_{i}\}_{n-1})  \end{array} $$

### 3off2 algorithm

The 3off2 scheme can be used to devise a two-step algorithm (see Algorithm 2), inspired by constraint-based approaches, to first reconstruct network skeleton (Algorithm 2, step 1) before combining orientation and propagation of edges in a single step based on likelihood ratios (Algorithm 2, step 2).

#### Reconstruction of network skeleton

The 3off2 scheme will first be applied to iteratively remove edges with *maximum positive contributions*, *I*^′^(*x*;*y*;*u*_*k*_|{*u*_*i*_}_*k*−1_)>0, corresponding to the *most likely generalized non-v-structures* (Eq. 23), while *minimizing simultaneously* the remaining 2-point information, *I*^′^(*x*;*y*|{*u*_*i*_}_*k*_) (Eq. 24), consistently with the data processing inequality. Such 3off2 scheme (Algorithm 2, step 1) will therefore progressively lower the conditional 2-point information terms, *I*^′^(*x*;*y*)>⋯>*I*^′^(*x*;*y*|{*u*_*i*_}_*k*−1_)>*I*^′^(*x*;*y*|{*u*_*i*_}_*k*_) and might ultimately result in the removal of the corresponding edge, *xy*, but only when a structural independency is actually found, *i.e.I*^′^(*x*;*y*|{*u*_*i*_}_*n*_)<0, as in constraint-based algorithms for a given significance level *α*. Yet, the skeleton obtained with the 3off2 scoring approach is expected to be more robust to finite observational data than the skeleton obtained with PC or IC algorithms, as the former results only from statistically significant 3-point contributions, *I*^′^(*x*;*y*;*u*_*k*_|{*u*_*i*_}_*k*−1_)>0, based on their quantitative 3off2 ranks, *R*(*xy*;*u*_*k*_|{*u*_*i*_}_*k*−1_).

The best results on benchmark networks using these quantitative 3off2 ranks are obtained with the NML score (see Results and discussion Section below). The MDL score leads to equivalent results, as expected, in the limit of very large datasets (see Appendix). However, with smaller datasets, the most reliable results with the MDL score are obtained using *non-shifted* instead of shifted 2-point and 3-point information terms in the 3off2 rank of individual edges, Eq. 30. This is because the MDL complexity tends to underestimate the importance of edges between nodes with many levels (see Appendix). For finite datasets, it easily leads to spurious conditional independencies, *I*^′^(*x*;*y*|{*ui*})<0, when using shifted 2-point and 3-point information, Eq. 33, whereas using non-shifted information in the 3off2 ranks (Eq. 30) tends to limit the number of false negatives as early errors in {*u*_*i*_} can only increase $I(x;y|\{ui\})\geqslant 0$, in the end, in Eq. 34.

#### Orientation of network skeleton

The skeleton and the separation sets resulting from the 3off2 iteration step (Algorithm 2, step 1) can then be used to orient the edges and to propagate orientations to the unshielded triples. However, while the constraint-based methods distinguish the v-structures orientation step (Algorithm 2, step 2) from the propagation procedure (Algorithm 1, step 3), the 3off2 algorithm intertwines these two steps based on the respective likelihood scores of individual v-structures and non-v-structures (Algorithm 2, step 2).

As stated earlier, the magnitude and sign of the conditional 3-point information, *I*(*x*;*y*;*z*|{*u*_*i*_}) (or equivalently the shifted 3-point information, Eq. 23), indicate if a non v-structure is more likely than a v-structure. Hence, all the unshielded triples can be ranked by the *absolute* value of their conditional 3-point information, that is, in decreasing order of their *likelihood* of being either a v-structure or a non-v-structure. As detailed in the step 2 of Algorithm 2, the most likely v-structure is used to set the first orientations, following *R*_0_ orientation rule. The possible propagations are then performed, following *R*_1_ propagation rule, starting from the unshielded triple having the most positive conditional 3-point information. The following most likely v-structure is considered when no further propagation is possible on unshielded triples with greater absolute 3-point information. If conflicting orientations arise (such as *a*→*b*←*c* & *b*→*c*←*d*), the less likely v-structure and its possible propagations are ignored.

Note that we only implement the *R*_0_ and *R*_1_ propagation rules, which are applied in decreasing order of likelihood. In particular, we do not consider propagation rules *R*_2_ and *R*_3_ which are not associated to likelihood scores but enforce the hypothesis of acyclic constraint.

As for the 3off2 skeleton reconstruction, the orientation/propagation step of 3off2 allows for a robust discovery of orientations from finite observational data as it relies on a quantitative framework of likelihood ratios taken in decreasing order of their statistical significance. During this step, 3off2 recovers and propagates as many orientations as possible in an iterative procedure following the decreasing ranks of the unshielded triples based on the absolute value of their conditional 3-point information, |*I*^′^(*x*;*y*;*z*|{*u*_*i*_})|.

## Results and discussion

### Tests on benchmark graphs

**Fig. 2:**
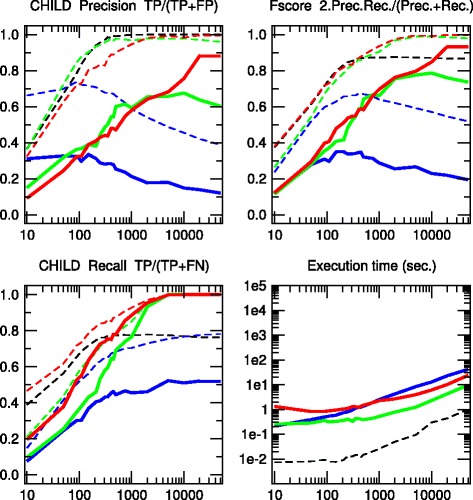
CHILD network. [20 nodes, 25 links, 230 parameters, Average degree 2.5, Maximum in-degree 2]. Precision, Recall and F-score for skeletons (*dashed lines*) and CPDAGs (*solid lines*). The results are given for Aracne (*black*), PC (*blue*), Bayesian Hill-Climbing (*green*) and 3off2 (*red*)

**Fig. 3:**
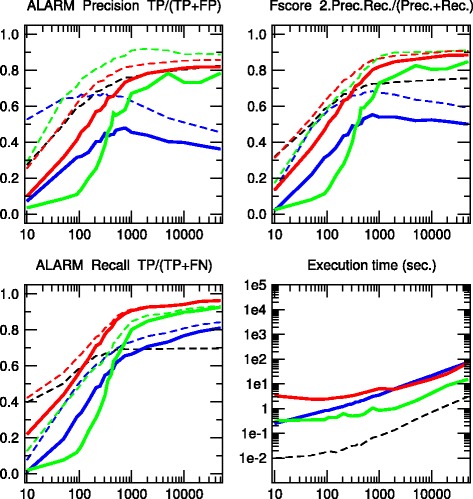
ALARM network. [37 nodes, 46 links, 509 parameters, Average degree 2.49, Maximum in-degree 4]. Precision, Recall and F-score for skeletons (*dashed lines*) and CPDAGs (*solid lines*). The results are given for Aracne (*black*), PC (*blue*), Bayesian Hill-Climbing (*green*) and 3off2 (*red*)

**Fig. 4:**
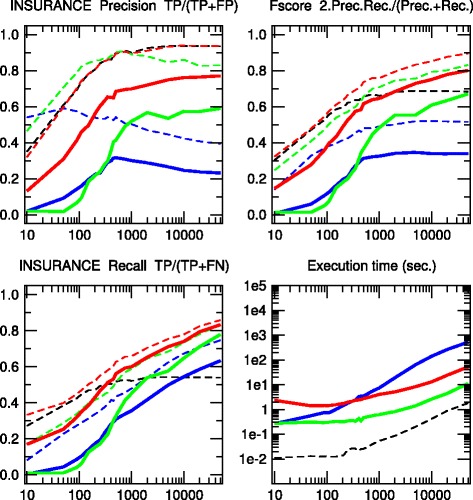
INSURANCE network. [27 nodes, 52 links, 984 parameters, Average degree 3.85, Maximum in-degree 3]. Precision, Recall and F-score for skeletons (*dashed lines*) and CPDAGs (*solid lines*). The results are given for Aracne (*black*), PC (*blue*), Bayesian Hill-Climbing (*green*) and 3off2 (*red*)

**Fig. 5:**
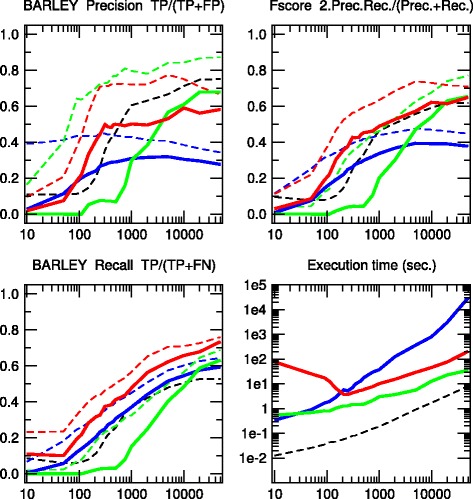
BARLEY network. [48 nodes, 84 links, 114,005 parameters, Average degree 3.5, Maximum in-degree 4]. Precision, Recall and F-score for skeletons (*dashed lines*) and CPDAGs (*solidlines*). The results are given for Aracne (*black*), PC (*blue*), Bayesian Hill-Climbing (*green*) and 3off2 (*red*)

**Fig. 6:**
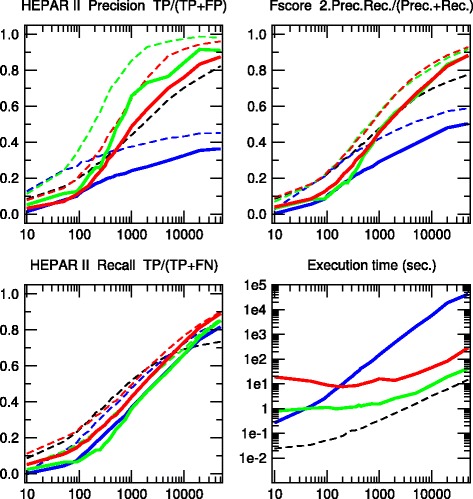
HEPAR II network. [70 nodes, 123 links, 1,453 parameters, Average degree 3.51, Maximum in-degree 6]. Precision, Recall and F-score for skeletons (*dashed lines*) and CPDAGs (*solid lines*). The results are given for Aracne (*black*), PC (*blue*), Bayesian Hill-Climbing (*green*) and 3off2 (*red*)



We have tested the 3off2 network reconstruction approach to learn benchmark causal graphs containing 20 to 70 nodes, Figs. 2, 3, 4, 5 and 6. The results are evaluated against other methods in terms of Precision (or positive predictive value), *Prec*=*TP*/(*TP*+*FP*), Recall or Sensitivity (true positive rate), *Rec*=*TP*/(*TP*+*FN*), as well as F-score =2×*Prec*×*Rec*/(*Prec*+*Rec*) for increasing sample size *N*=10 to 50,000 data points.

We also define additional Precision, Recall and F-scores taking into account the edge orientations of the predicted networks against the corresponding CPDAG of the benchmark networks. This amounts to label as false positives, all true positive edges of the skeleton with different orientation/non-orientation status as the CPDAG reference, *TP*_misorient_, leading to the orientation-dependent definitions *TP*^′^=*TP*−*TP*_misorient_ and *FP*^′^=*FP*+*TP*_misorient_ with the corresponding CPDAG Precision, Recall and F-scores taking into account edge orientations.

The alternative inference methods used for comparison with 3off2 are the PC algorithm [12] implemented in the pcalg package [25, 26] and Bayesian inference using the hill-climbing heuristics implemented in the bnlearn package [27]. In addition, we also compare the skeleton of 3off2 to the unoriented output of Aracne [28], an information-based inference approach, which iteratively prunes links with the weakest mutual information based on the Data Processing Inequality. We have used the Aracne implementation of the minet package [29]. For each sample size, 3off2, Aracne, PC and the Bayesian inference methods have been tested on 50 replicates. Figures 2, 3, 4, 5 and 6 give the average results over these multiple replicates when comparing the CPDAG (solid lines) of the reconstructed network (or its skeleton, dashed lined) to the CPDAG (or the skeleton) of the benchmark network.

For each method, the plots presented in Figs. 2, 3, 4, 5 and 6 are those obtained for the parameters that give overall the best results over the five reconstructed benchmark networks (see Additional file 1, Figures S1-S20). In particular, we used the *stable* implementation of the PC algorithm, as well as the *majority rule* for the orientation and propagation steps [14]. PC’s results are shown on Figs. 2, 3, 4, 5 and 6 for *α*=0.1. Decreasing *α* tends to improve the skeleton Precision at the expense of the skeleton Recall, leading in fact to worse skeleton F-scores for finite datasets, *e.g.N*≤1000 (see Additional file 1, Figures S1-S5). The same trend is observed for CPDAG F-scores taking into account edge orientations, with best CPDAG scores at small sample sizes, obtained for larger *α*, *e.g.N*≤1000. Aracne threshold parameters for minimum difference in mutual information is set to *ε*=0, as small positive values typically worsen F-scores (see Additional file 1, Figures S6-S10). Bayesian inference are obtained using BIC/MDL scores and hill-climbing heuristics with 100 random restarts [9] (see Additional file 1, Figures S11-S15). Finally, the best 3off2 network reconstructions are obtained using NML scores with shifted 2-point and 3-point information terms in the rank of individual edges, see Methods. Using MDL scores, instead, leads to equivalent results, as expected, in the limit of very large datasets (see Appendix). However, with smaller datasets, the most reliable results with MDL scores are obtained using *non-shifted* instead of shifted 2-point and 3-point information terms in the 3off2 rank of individual edges, as discussed in Methods (see Additional file 1, Figures S16-S20).

All in all, we found that the 3off2 inference approach typically reaches better or equivalent F-scores for all dataset sizes as compared to all other tested methods, *i.e.* Aracne, PC and Bayesian inference, as well as the Max-Min Hill-Climbing (MMHC) hybrid method [30] (see Additional file 1, Figures S21-S25). This is clearly observed both on the skeletons (Figs. 2, 3, 4, 5 and 6 dashed lines) and even more clearly when taking the predictions of orientations into account (Figures 2, 3, 4, 5 and 6 solid lines).

### Applications to the hematopoiesis regulation network

The reconstruction or reverse-engineering of real regulatory networks from actual expression data has already been performed on a number of biological systems (see *e.g.* [28, 31–33]). Here, we apply the 3off2 approach on a real biological dataset related to hematopoiesis. Transcription factors play a central role in hematopoiesis, from which derive the blood cell lineages. As suggested in previous studies, changes in the regulatory interactions among transcription factors [34] or their overexpression [35] might be involved in the development of T-acute lymphoblastic leukaemia (T-ALL). The key role of the hematopoiesis and the potentially serious consequences of its disregulations emphasize the need to accurately establish the complex interactions between the transcription factors involved in this critical biological process.

**Fig. 7:**
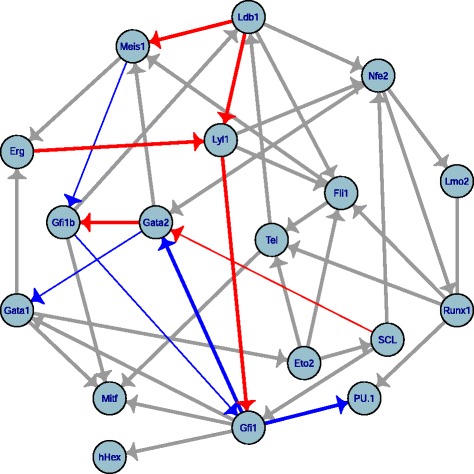
Hematopoietic subnetwork reconstructed by 3off2. The dataset [36] concerns 18 transcription factors, 597 single cells, 5 different hematopoietic progenitor types. Red and blue edges correspond to experimentally proven activations and repressions, respectively as reported in the literature (Table 1), while grey links indicate regulatory interactions for which no clear evidence has been established so far. Thinner arrows underline 3off2 misorientations

The dataset we have used for this analysis [36] consists of the single cell expressions of 18 transcription factors, known for their role in hematopoiesis. Five hundred ninety seven single cells representing 5 different types of hematopoietic progenitors have been included in the analysis (*N*=597). We reconstructed the corresponding network with the 3off2 inference method, Fig. 7, and four other available approaches, namely, PC [12] implemented in the pcalg package [25, 26], Bayesian inference using hill-climbing heuristics as well as the Max-Min Hill-Climbing (MMHC) hybrid method [30], both implemented in the bnlearn package [27], and, finally, Aracne [28] implemented in the minet package [29] (Table 1 and Additional file 1: Table S1).

**Table 1:** Interactions reconstructed by 3off2 and alternative methods for a subnetwork of hematopoiesis regulation. → indicates a successfully recovered interaction including its direction as reported in the literature (see References). ${\nrightarrow }$ corresponds to a successfully recovered interaction, however, with an opposite direction as reported in the literature. ⌿ stipulates that no direct regulatory interaction has been inferred, while — corresponds to an undirected link. Note in particular that Aracne does not infer edge direction. See Additional file 1: Table S1 for supplementary statistics

11 known Regulatory		3off2	PC	PC	MMHC	MMHC	Bayes hc	Bayes hc	Aracne
interactions	References	*NML*	*α* * = 10* ^*−1*^	*α* * = 10* ^*−2*^	*BDe*	*BIC*	*BDe*	*BIC*	*ε=0*
Gata2 → Gfi1b	[36]	→	${\nrightarrow }$	—	⌿	⌿	→	⌿	⌿
Gfi1 → Gata2	[36]	→	→	—	→	${\nrightarrow }$	→	${\nrightarrow }$	—
Gfi1b ${\leftrightarrows }$ Gfi1	[36]	${\nrightarrow }$	${\nrightarrow }$	—	${\nrightarrow }$	${\nrightarrow }$	${\nrightarrow }$	${\nrightarrow }$	—
Gfi1 → PU.1	[37]	→	→	⌿	⌿	⌿	→	→	—
Lyl1 → Gfi1	[38]	→	${\nrightarrow }$	⌿	⌿	⌿	→	${\nrightarrow }$	—
Ldb1 → Meis1	[39]	→	⌿	⌿	⌿	⌿	${\nrightarrow }$	⌿	⌿
Ldb1 → Lyl1	[39]	→	⌿	⌿	⌿	⌿	⌿	⌿	⌿
Erg → Lyl1	[40]	→	${\nrightarrow }$	—	→	→	→	${\nrightarrow }$	—
Gata2 → Scl	[40]	${\nrightarrow }$	→	—	→	→	→	→	—
Gfi1b → Meis1	[41]	${\nrightarrow }$	${\nrightarrow }$	—	→	→	→	→	—
Gata1 → Gata2	[42]	${\nrightarrow }$	${\nrightarrow }$	—	→	→	→	→	—
Correct edges (out of **11**)	(→/${\nrightarrow }$/—)	**11**	**9**	**7**	**6**	**6**	**10**	**8**	**8**
- Correct orientations	(→)	7	3	0	5	4	8	4	0
- Mis/non-orientations	(${\nrightarrow }$/ —)	4	6	7	1	2	2	4	8
Missing links	(⌿)	**0**	**2**	**4**	**5**	**5**	**1**	**3**	**3**

3off2 uncovers all 11 interactions for which specific experimental evidence has been reported in the literature (Fig. 7, red links: known activations; blue links: known repressions) as well as 30 additional links (Fig. 7, grey links: unknown regulatory interactions). By contrast, randomization of the actual data across samples for each TF leads to only 5.25 spurious interactions on average between the 18 TFs, instead of the 41 inferred edges from the actual data, and 1.62 spurious interactions on average, instead of the 16 interactions predicted among the 10 TFs involved in known regulatory interactions, Fig. 7. This suggests that around 10–13 % of the predicted edges might be spurious, due to inevitable sampling noise in the finite dataset. In particular, the 3off2 inference approach successfully recovers the relationships of the regulatory triad between *Gata2*, *Gfi1b* and *Gfi1* as described in [36] and reports correct orientations for the edges involving *Gata2* (*Gfi1b* and *Gfi1* crossregulate in fact one another [36], Table 1). The network reconstructed by 3off2 also correctly infers the regulations of *PU.1* by *Gfi1* [37], *Gfi1* by *Lyl1* [38], *Meis1* by *Ldb1* [39], and the regulations of *Lyl1* by *Ldb1* [39] and *Erg* [40]. Finally, the interactions (*Gata2* −*SCL*) [40], (*Gfi1b* −*Meis1*) [41] and (*Gata1* −*Gata2*) [42] are correctly inferred, however, with opposite directions as reported in the literature. Yet, overall 3off2 outperforms most of the other methods tested for the reconstruction of the hematopoietic regulatory subnetwork (Table 1 and Additional file 1: Table S1). Only the Bayesian hill-climbing method using a BDe score leads to comparable results by retrieving 10 out of 11 interactions and correctly orienting 8 of them. These encouraging results from the 3off2 reconstruction method on experimentally proven regulatory interactions (red edges in Fig. 7) could motivate further investigations on novel regulatory interactions awaiting to be tested for their possible role in hematopoiesis (*e.g.* grey edges in Fig. 7).

## Conclusions

In this paper, we propose to improve constraint-based network reconstruction methods by identifying structural independencies through a robust quantitative score-based scheme limiting the accumulation of early FN errors and subsequent FP compensatory errors. In brief, 3off2 relies on information theoretic scores to progressively uncover the best supported conditional independencies, by iteratively “taking off” the most likely indirect contributions of conditional 3-point information from every 2-point (mutual) information of the causal graph.

Earlier hybrid methods have also attempted to improve network reconstruction by combining the concepts of constraint-based approaches with the robustness of Bayesian scores [30, 43–45]. In particular [43], have proposed to exploit an intrinsic weakness of the PC algorithm, its sensitivity to the order in which conditional independencies are tested on finite data, to rank these different order-dependent PC predictions with Bayesian scores. More recently [30], have also combined constraint-based and Bayesian approaches by first identifying both parents and children of each node of the underlying graphical model and then performing a greedy Bayesian hill-climbing search restricted to the identified parents and children of each node. This Max-Min Hill-Climbing (MMHC) approach tends to have a high precision in terms of skeleton but a more limited sensibility, leading overall to lower skeleton and CPDAG F-scores than 3off2 and Bayesian hill climbing methods on the same benchmark networks, Figures S21-S25. Interestingly, however, the MMHC approach is among the fastest network reconstruction approaches, Figure S26, allowing for scalability to large network sizes [30].

The 3off2 algorithm is expected to run in polynomial time on *typical* sparse causal networks with low in-degree, just like constraint-based algorithms.However, in practice and despite the additional computation of conditional 2-point and 3-point information terms, we found that the 3off2 algorithm runs typically faster than constraint-based algorithms for large enough samples, by avoiding the cascading accumulation of errors that inflate the combinatorial search of conditional independencies in traditional constraint-based approaches. Instead, we found that 3off2 running time displays a similar trend as Bayesian hill-climbing heuristic methods, Figs. 2, 3, 4, 5 and 6.

All in all, the main computational bottleneck of the present 3off2 scheme pertains to the identification of the *best* contributing nodes at each iteration. In the future, it could be interesting to investigate whether a more stochastic version of this 3off2 method, based on choosing *one* significant conditional 3-point information instead of the best one, might simultaneously accelerate the network reconstruction and circumvent possible locally trapped suboptimal predictions through stochastic resampling.

Finally, another perspective for practical applications will be to include the possibility of latent variables and bidirected edges in reconstructed networks.

## Appendix

### Complexity of graphical models

The complexity $k_{\mathcal {G,D}}$ of a graphical model is related to the normalization constant $Z(\mathcal {G,D})$ of its maximum likelihood as $k_{\mathcal {G,D}}=\log Z(\mathcal {G,D})$,

(35) $$\begin{array}{*{20}l} {\mathcal{L_{G}}}&={e^{-N H(\mathcal{G,D})}\over Z(\mathcal{G,D})}={e^{-N H(\mathcal{G,D})-k_{\mathcal{G,D}}}}  \end{array} $$

For Bayesian networks with decomposable entropy, *i.e.*$H(\mathcal {G,D})=\sum _{i} H(x_{i}\vert \{\text {Pa}_{x_{i}}\})$, it is convenient to use decomposable complexities, $k_{\mathcal {G,D}}=\sum _{i} k_{x_{i}\vert \left \{\text {Pa}_{x_{i}}\right \}}$,

(36) $$\begin{array}{*{20}l} {\mathcal{L_{G}}}&={e^{-N \sum_{i} H\left(x_{i}\vert \left\{\text{Pa}_{x_{i}}\right\}\right) -\sum_{i} k_{x_{i}\vert \left\{\text{Pa}_{x_{i}}\right\}} }}  \end{array} $$

such that the comparison between alternative models $\mathcal {G}$ and $\mathcal {G}_{\setminus {x\to y}}$ (*i.e.*$\mathcal {G}$ with one missing edge *x*→*y*) leads to a simple local increment of the score,

(37) $$\begin{array}{*{20}l} {\mathcal{L}_{\mathcal{G}_{\setminus{x\to y}}}\over \mathcal{L}_{\mathcal{G}}}&={e^{-N I(x;y\vert \{\text{Pa}_{y}\}_{\setminus{x}}) +\Delta k_{y \vert \left\{\text{Pa}_{y}\right\}_{\setminus{x}}} }} \end{array} $$

(38) $$\begin{array}{*{20}l} I(x;y\vert \{\text{Pa}_{y}\}_{\setminus{x}})&=H(y\vert \{\text{Pa}_{y}\}_{\setminus{x}})-H(y\vert \{\text{Pa}_{y}\})\geqslant 0 \end{array} $$

(39) $$\begin{array}{*{20}l} \Delta k_{y\vert \{\text{Pa}_{y}\}_{\setminus{x}}}&=k_{y\vert \{\text{Pa}_{y}\}}-k_{y\vert \{\text{Pa}_{y}\}_{\setminus{x}}}\geqslant 0 \end{array} $$

A common complexity criteria in model selection is the Bayesian Information Criteria (BIC) or Minimal Description Length (MDL) criteria [19, 20],

(40) $$\begin{array}{*{20}l} k^{^{\sf MDL}}_{y\vert \{\text{Pa}_{y}\}}&={1\over 2}(r_{y}-1)\prod^{\text{Pa}_{y}}_{j} r_{j} \;\log N \end{array} $$

(41) $$\begin{array}{*{20}l} \Delta k^{^{\sf MDL}}_{y\vert \{\text{Pa}_{y}\}_{\setminus{x}}}&={1\over 2}(r_{x}-1)(r_{y}-1)\prod^{\text{Pa}_{y_{\setminus{x}}}}_{j} r_{j} \;\log N \end{array} $$

where *r*_*x*_,*r*_*y*_ and *r*_*j*_ are the number of levels of each variable, *x*, *y* and *j*. The MDL complexity, Eq. 40, is simply related to the normalisation constant reached in the asymptotic limit of a large dataset *N*→*∞* (Laplace approximation). The MDL complexity can also be derived from the Stirling approximation on the Bayesian measure [46, 47]. Yet, in practice, this limit distribution is only reached for very large datasets, as some of the least-likely $(r_{y}-1)\prod _{j} r_{j}$ combinations of states of variables are in fact rarely (if ever) sampled in typical finite datasets. As a result, the MDL complexity criteria tends to underestimate the relevance of edges connecting variables with many levels, *r*_*i*_, leading to the removal of false negative edges.

To avoid such biases with finite datasets, the normalisation of the maximum likelihood can be done over all possible datasets with the same number *N* of data points. This corresponds to the (universal) Normalized Maximum Likelihood (NML) criteria [21–24],

(42) $$\begin{array}{*{20}l} {\mathcal{L_{G}}}&={e^{-N H(\mathcal{G,D})}\over \sum_{\vert\mathcal{D'}\vert=N}e^{-N H(\mathcal{G,D'})}}={e^{-N H(\mathcal{G,D})-k^{^{\sf NML}}_{\mathcal{G,D}}}}  \end{array} $$

We introduce here the factorized version of the NML criteria [23, 24] which corresponds to a decomposable NML score, $k^{^{\sf NML}}_{\mathcal {G,D}}=\sum _{x_{i}} k^{^{\sf NML}}_{x_{i}\vert \{\text {Pa}_{x_{i}}\}}$, defined as,

(43) $$\begin{array}{*{20}l} k^{^{\sf NML}}_{y\vert \{\text{Pa}_{y}\}}&=\sum^{q_{y}}_{j} \log {\mathcal{C}^{r_{y}}_{N_{yj}}} \end{array} $$

(44) $$\begin{array}{*{20}l} \Delta k^{^{\sf NML}}_{y\vert \{\text{Pa}_{y}\}_{\setminus{x}}}&=\sum^{q_{y}}_{j} \log {\mathcal{C}^{r_{y}}_{N_{yj}}}-\sum^{q_{y}/r_{x}}_{j'} \log {\mathcal{C}^{r_{y}}_{N_{yj'}}}  \end{array} $$

where *N*_*yj*_ is the number of data points corresponding to the *j*th state of the parents of *y*, {Pa_*y*_}, and $\phantom {\dot {i}\!}N_{yj^{\prime }}$ the number of data points corresponding to the *j*^′^th state of the parents of *y*, excluding *x*, {Pa_*y*_}_∖*x*_. Hence, the factorized NML score for each node *x*_*i*_ corresponds to a separate normalisation for each state *j*=1,…,*q*_*i*_ of its parents and involving exactly *N*_*ij*_ data points of the finite dataset,

(45) $$\begin{array}{*{20}l} {\mathcal{L_{G}}}&={e^{-N \sum_{i} H(x_{i}\vert \{\text{Pa}_{x_{i}}\}) -{{\sum_{i}}}\sum^{q_{i}}_{j} \log {\mathcal{C}^{r_{i}}_{N_{ij}}}}} \end{array} $$

(46) $$\begin{array}{*{20}l} &={e^{N \sum_{i} \sum^{q_{i}}_{j} \sum^{r_{i}}_{k} {N_{ijk}\over N}\log\left({N_{ijk}\over N_{ij}}\right) -{{\sum_{i}}}\sum^{q_{i}}_{j} \log {\mathcal{C}^{r_{i}}_{N_{ij}}}}} \end{array} $$

(47) $$\begin{array}{*{20}l} &=\prod_{i} \prod^{q_{i}}_{j} {\prod^{r_{i}}_{k} \left({N_{ijk}\over N_{ij}}\right)^{N_{ijk}}\over {\mathcal{C}^{r_{i}}_{N_{ij}}}}  \end{array} $$

where *N*_*ijk*_ corresponds to the number of data points for which the *i*th node is in its *k*th state and its parents in their *j*th state, with $N_{\textit {ij}}=\sum ^{r_{i}}_{k} N_{\textit {ijk}}$. The universal normalization constant ${\mathcal {C}^{r}_{n}}$ is then obtained by averaging over all possible partitions of the *n* data points into a maximum of *r* subsets, *ℓ*_1_+*ℓ*_2_+⋯+*ℓ*_*r*_=*n* with $\ell _{k}\geqslant 0$,

(48) $$\begin{array}{*{20}l} {\mathcal{C}^{r}_{n}}&=\sum_{\ell_{1}+\ell_{2}+\cdots+\ell_{r}=n} {{n}!\over{{\ell_{1}}!{\ell_{2}}!\cdots{\ell_{r}}!}}\prod^{r}_{k=1}\left({\ell_{k}\over n}\right)^{\ell_{k}}  \end{array} $$

which can in fact be computed in linear-time using the following recursion [23],

(49) $$\begin{array}{*{20}l} {\mathcal{C}^{r}_{n}}&={\mathcal{C}^{r-1}_{n}} + {n\over{r-2}} {\mathcal{C}^{r-2}_{n}}  \end{array} $$

with ${\mathcal {C}^{r}_{0}}=1$ for all *r*, ${\mathcal {C}^{1}_{n}}=1$ for all *n* and applying the general formula Eq. 48 for *r*=2,

(50) $$\begin{array}{*{20}l} {\mathcal{C}^{2}_{n}}&=\sum^{n}_{h=0} \binom{n}{h} \left({h\over n}\right)^{h} \left({{n-h}\over n}\right)^{n-h}  \end{array} $$

or its Szpankowski approximation for large *n* (needed for *n*>1000 in practice) [48–50],

(51) $$\begin{array}{*{20}l} {\mathcal{C}^{2}_{n}}&=\sqrt{n\pi \over 2} \left(1+{2\over 3}\sqrt{2\over{n\pi}}+{1\over{12n}}+\mathcal{O}\left({1 \over n^{3/2}}\right)\right) \end{array} $$

(52) $$\begin{array}{*{20}l} &\simeq\sqrt{n\pi \over 2} \exp\left({\sqrt{8\over{9n\pi}}+{{3\pi -16}\over{36n\pi}}}\right)  \end{array} $$

Then, following the rationale of constraint-based approaches, we can reformulate the likelihood ratio of Eq. 37 by replacing the parent nodes {Pa_*y*_}_∖*x*_ in the conditional mutual information, *I*(*x*;*y*|{Pa_*y*_}_∖*x*_), with an unknown separation set {*u*_*i*_} to be learnt simultaneously with the missing edge candidate *xy*,

(53) $$\begin{array}{*{20}l} {\mathcal{L}_{\mathcal{G}_{\setminus{xy\vert \{u_{i}\}}}}\over \mathcal{L}_{\mathcal{G}}}&= {e^{-N I(x;y\vert \{u_{i}\})+ k_{x;y\vert \{u_{i}\}}}}  \end{array} $$

where we have also transformed the asymmetric parent-dependent complexity difference, $\Delta k_{y\vert \{\phantom {\dot {i}\!}\text {Pa}_{y}\}_{\setminus {x}}}$, into a {*u*_*i*_}-dependent complexity term, $k_{x;y\vert \{u_{i}\}}$, with the same *xy*-symmetry as *I*(*x*;*y*|{*u*_*i*_}),

(54) $$\begin{array}{*{20}l} k^{^{\sf MDL}}_{x;y\vert \{u_{i}\}}&={1\over 2}(r_{x}-1)(r_{y}-1)\prod_{i}r_{u_{i}}\log N \end{array} $$

(55) $$\begin{array}{*{20}l} k^{^{\sf NML}}_{x;y\vert \{u_{i}\}}&={1\over 2}\sum^{\{u_{i}\}}_{j'} \Big(\sum^{r_{x}}_{k_{x}} \log {\mathcal{C}^{r_{y}}_{N_{k_{x}j'}}}- \log {\mathcal{C}^{r_{y}}_{N_{j'}}}\\ &\;\;\;\;\;\;\;\;\;\;\;\;\, +\sum^{r_{y}}_{k_{y}} \log {\mathcal{C}^{r_{x}}_{N_{k_{y}j'}}}- \log {\mathcal{C}^{r_{x}}_{N_{j'}}} \Big) \end{array} $$

Note, in particular, that the MDL complexity term in Eq. 54 is readily obtained from Eq. 41 due to the Markov equivalence of the MDL score, corresponding to its *xy*-symmetry whenever {Pa_*y*_}_∖*x*_={Pa_*x*_}_∖*y*_. By contrast, the factorized NML score, Eq. 43, is not a Markov-equivalent score (although its non-factorized version, Eq. 42, is Markov equivalent by definition). To circumvent this non-equivalence of factorized NML score, we propose to recover the expected *xy*-symmetry of $k^{^{\sf NML}}_{x;y\vert \{u_{i}\}}$ through the simple *xy*-symmetrization of Eq. 44, leading to Eq. 55.

## Additional file

Additional file 1
**Complementary evaluations for the** 3off2 **inference approach and comparisons with alternative reconstruction methods and parameters values.** In this additional file, the results of the 3off2 inference approach are evaluated against other methods in terms of Precision (or positive predictive value), *P* *r* *e* *c*=*T* *P*/(*T* *P*+*F* *P*), Recall or Sensitivity (true positive rate), *R* *e* *c*=*T* *P*/(*T* *P*+*F* *N*), as well as F-score =2×*P* *r* *e* *c*×*R* *e* *c*/(*P* *r* *e* *c*+*R* *e* *c*) and execution time when comparing the CPDAG of the reconstructed network (or its skeleton) to the CPDAG (or the skeleton) of the benchmark network. The alternative methods are the PC algorithm, the Bayesian inference method using the hill-climbing heuristics, the Max-Min Hill-Climbing (MMHC) hybrid method and the Aracne inference approach. (PDF 528 KB)
