# Supplementary material for: 3off2: A network reconstruction algorithm based on 2-point and 3-point information statistics
Source: BMC Bioinformatics. 2016 Jan 20;17(Suppl 2):12. doi: 10.1186/s12859-015-0856-x (PMC4959376; doi:10.1186/s12859-015-0856-x)
Supplement: Additional file 1 — Complementary evaluations for the 3off2 inference approach and comparisons with alternative reconstruction methods and parameters values. In this additional file, the results of the 3off2 inference approach are evaluated against other methods in terms of Precision (or positive predictive value), P r e c=T P/(T P+F P), Recall or Sensitivity (true positive rate), R e c=T P/(T P+F N), as well as F-score =2×P r e c×R e c/(P r e c+R e c) and execution time when comparing the CPDAG of the reconstructed network (or its skeleton) to the CPDAG (or the skeleton) of the benchmark network. The alternative methods are the PC algorithm, the Bayesian inference method using the hill-climbing heuristics, the Max-Min Hill-Climbing (MMHC) hybrid method and the Aracne inference approach. (PDF 528 KB) [file 12859_2015_856_MOESM1_ESM.pdf]

Additional File 1

3off2: a network reconstruction algorithm based on 2-point and 3-point information statistics

Séverine Affeldt   Louis Verny   Hervé Isambert*
CNRS UMR168, UPMC, Institut Curie, Research Center
26 rue d'Ulm, 75005 Paris, France

`severine.affeldt | louis.verny | herve.isambert @curie.fr`

In this additional file, the results of the network reconstruction methods considered in the main text are evaluated for different parameter values and against each others in terms of Precision (or positive predictive value), $Prec = TP/(TP + FP)$, Recall or Sensitivity (true positive rate), $Rec = TP/(TP+FN)$, as well as F-score = $2\times Prec \times Rec/(Prec+Rec)$ and execution time. The alternative methods are:

- the PC algorithm [1] with stable skeleton implementation and majority rule orientations [2], as implemented in the `pcalg` package [3, 4]
- the Bayesian inference method using the hill-climbing heuristics implemented in the `bnlearn` package [5]
- Aracne [6], an information-based inference approach, which iteratively prunes links with the weakest mutual information based on the Data Processing Inequality. We have used the Aracne implementation of the `minet` package [7]
- the MMHC algorithm: an hybrid approach [8] combining constraint-based and Bayesian approaches by first identifying both parents and children of each node of the underlying graphical model and then performing a greedy Bayesian hill-climbing search restricted to the identified parents and children of each node. The MMHC method is implemented in the `bnlearn` package [5].

Comparisons are made between the reconstructed network (or its CPDAG for Bayesian and MMHC methods) and the CPDAG of the benchmark network (filled line). TP with incorrect orientation or incorrect non-orientation are counted as FP edges (see main text). In addition, the skeleton of the reconstructed network is also compared to the skeleton of the benchmark network (dashed line). Note that Aracne only predicts network skeletons.

*Contact author

For sample sizes from $N = 10$ to $50,000$ data points, the methods have been tested on 50 replicates and the Figures S1-S26 give the average results over these multiple replicates. The five following benchmark networks have been considered (refer to the `bnlearn` package [5] for more details on these networks):

CHILD 20 nodes, 25 links, 230 parameters, average degree 2.5, maximum in-degree 2

INSURANCE 27 nodes, 52 links, 984 parameters, average degree 3.85, maximum in-degree 3

ALARM 37 nodes, 46 links, 509 parameters, average degree 2.49, maximum in-degree 4

BARLEY 48 nodes, 84 links, 114,005 parameters, average degree 3.5, maximum in-degree 4

HEPAR II 70 nodes, 123 links, 1,453 parameters, average degree 3.51, maximum in-degree 6

1 Evaluation of the PC method by significance level

In this section, the results of the PC inference method [1], as implemented in the `pcalg` package [3, 4], are evaluated using the following parameter values:

- a significance level $\alpha = 0.001$ (**PC 1e-03**)
- a significance level $\alpha = 0.01$ (**PC 1e-02**)
- a significance level $\alpha = 0.1$ (**PC 1e-01**)

In particular, the *stable* implementation of the PC algorithm has been used, as well as the *majority rule* for the orientation and propagation steps [2]. As shown in Figures S1-S5, the PC inference method typically requires the significance level to be adjusted to larger values ($\alpha = 0.1$) at small sample sizes and to smaller values ($\alpha = 0.01$ or $\alpha = 0.001$) on larger datasets to improve the CPDAG F-scores.

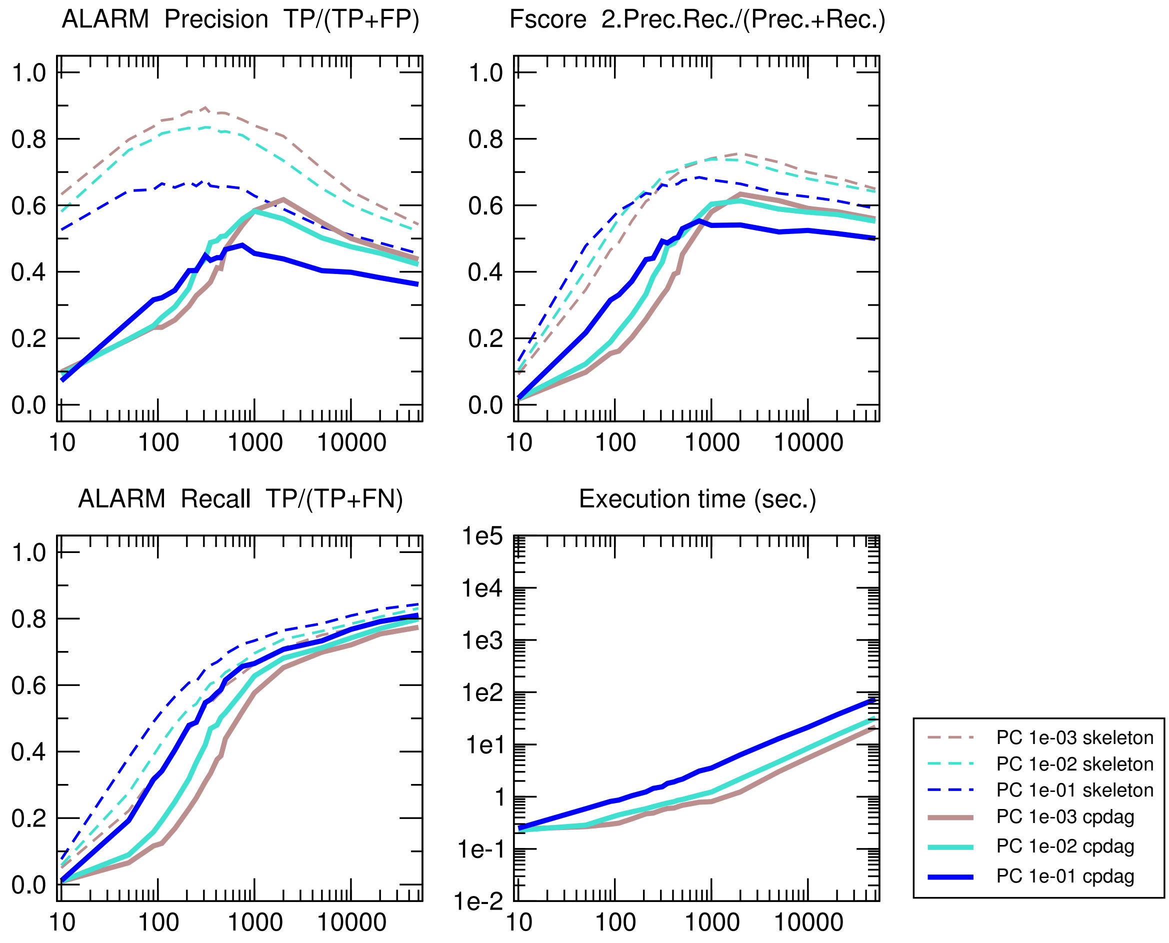

Figure S1: **ALARM network** [37 nodes, 46 links, 509 parameters, Average degree 2.49, Maximum in-degree 4]. Precision, Recall, F-score and execution time for the reconstruction of the skeletons (dashed lines) and CPDAGs (filled lines) using the PC inference approach.

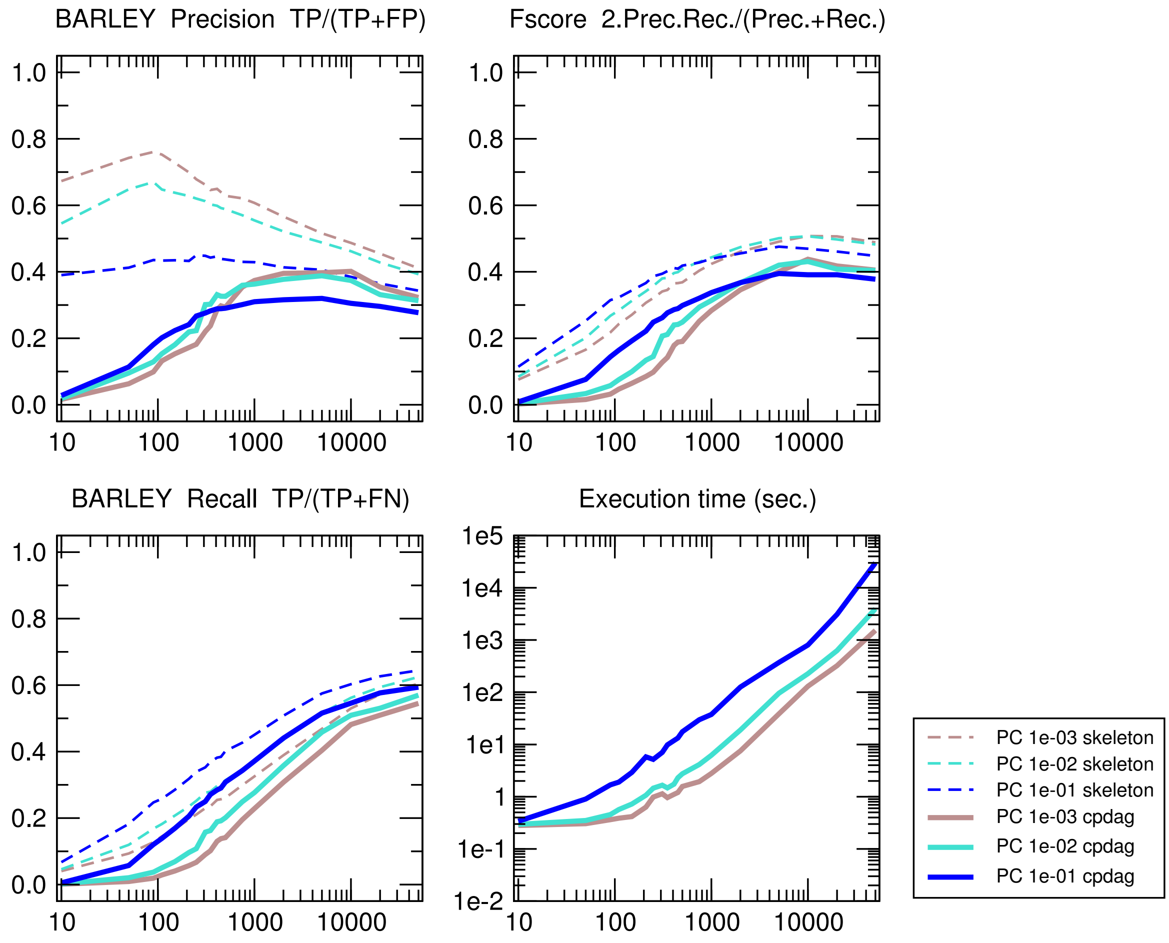

Figure S2: **BARLEY network** [48 nodes, 84 links, 114,005 parameters, Average degree 3.5, Maximum in-degree 4]. Precision, Recall, F-score and execution time for the reconstruction of the skeletons (dashed lines) and CPDAGs (filled lines) using the PC inference approach.

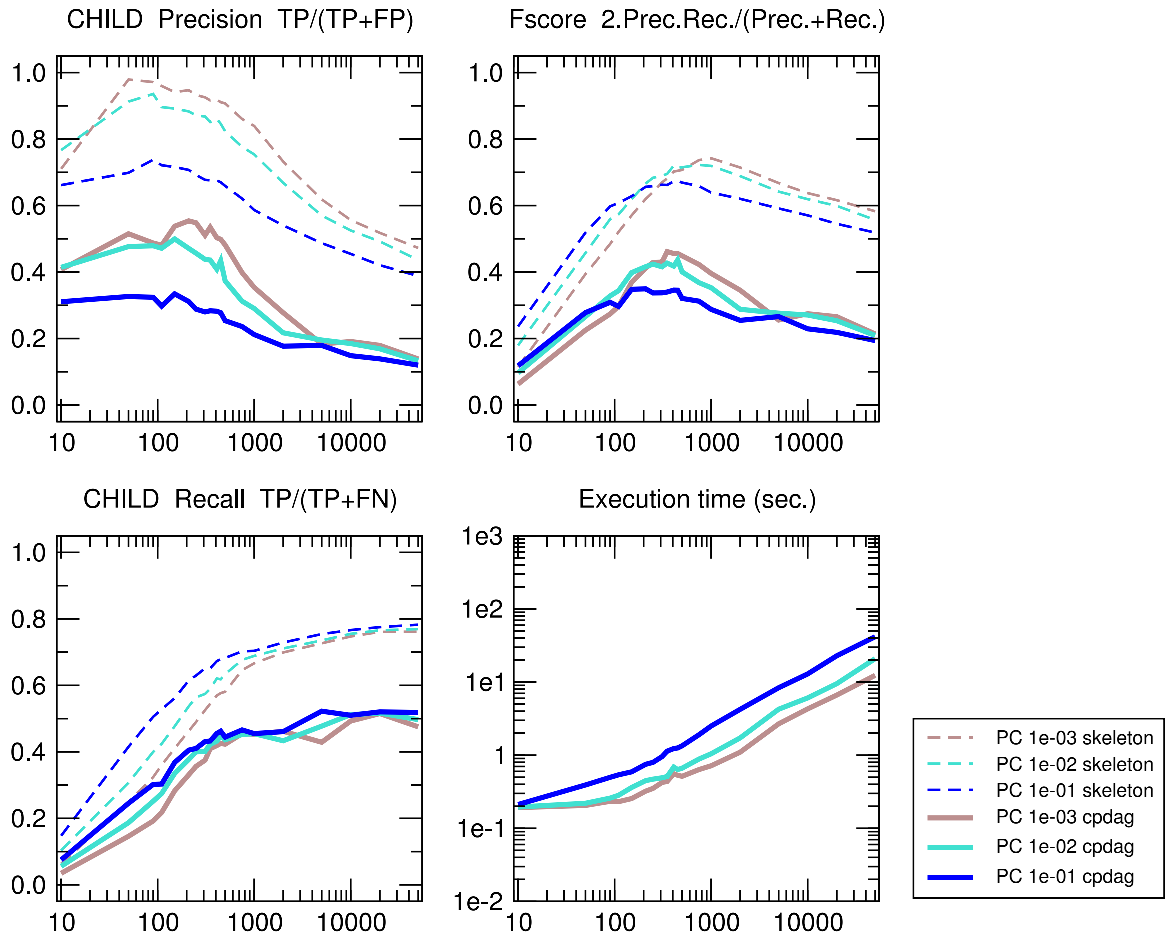

Figure S3: **CHILD network** [20 nodes, 25 links, 230 parameters, Average degree 2.5, Maximum in-degree 2]. Precision, Recall, F-score and execution time for the reconstruction of the skeletons (dashed lines) and CPDAGs (filled lines) using the PC inference approach.

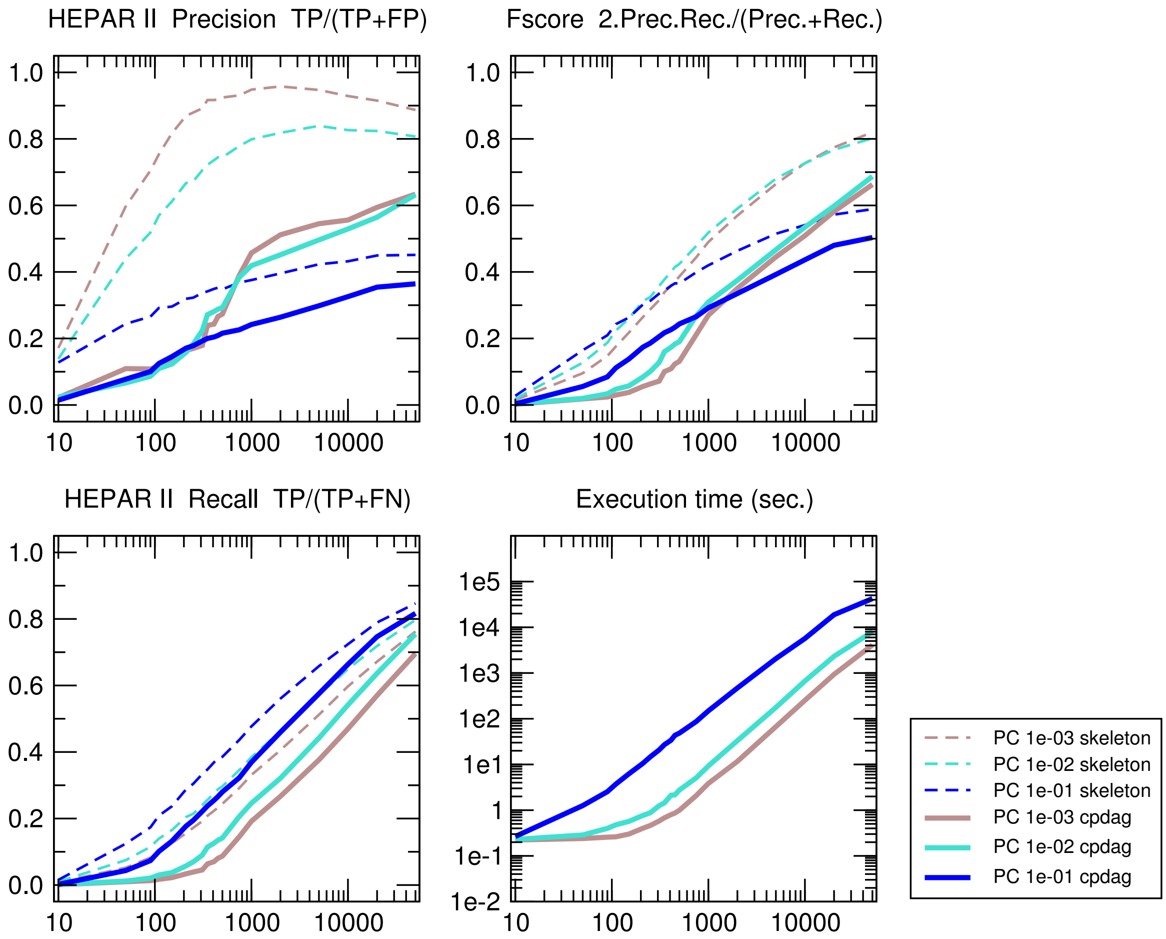

Figure S4: **HEPAR II network** [70 nodes, 123 links, 1,453 parameters, Average degree 3.51, Maximum in-degree 6]. Precision, Recall, F-score and execution time for the reconstruction of the skeletons (dashed lines) and CPDAGs (filled lines) using the PC inference approach.

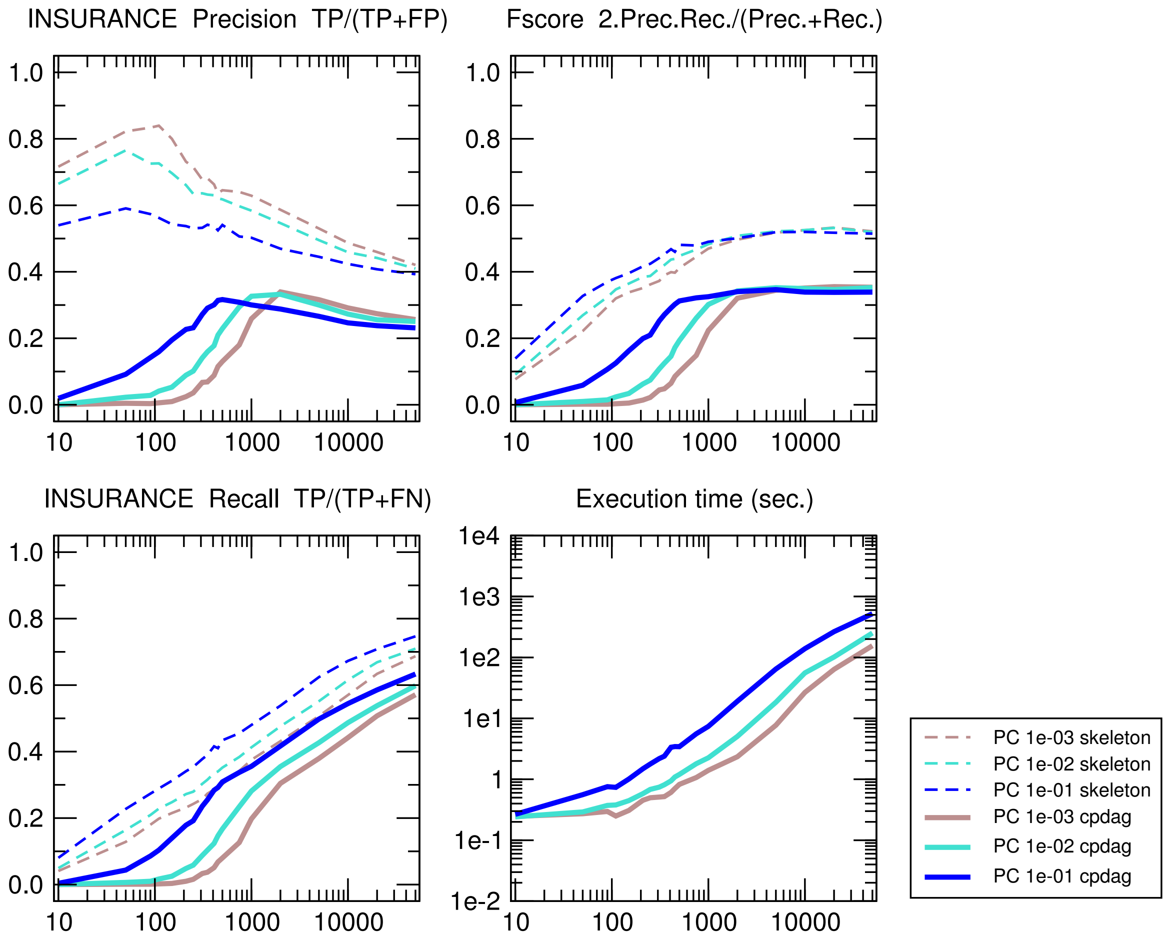

Figure S5: **INSURANCE network** [27 nodes, 52 links, 984 parameters, Average degree 3.85, Maximum in-degree 3]. Precision, Recall, F-score and execution time for the reconstruction of the skeletons (dashed lines) and CPDAGs (filled lines) using the PC inference approach.

2 Evaluation of the Aracne reconstruction method

In this section, the results of the Aracne inference method, as implemented in the `minet` package [7], are evaluated using the following parameter values:

- a threshold for the minimum difference in mutual information set to $\epsilon = 1/N$ (**Aracne Eps 1/N**)
- a threshold for the minimum difference in mutual information set to $\epsilon = 0$ (**Aracne Eps 0**)

As shown in Figures S6-S10, small positive values for the threshold parameters for minimum difference in mutual information typically worsen F-scores.

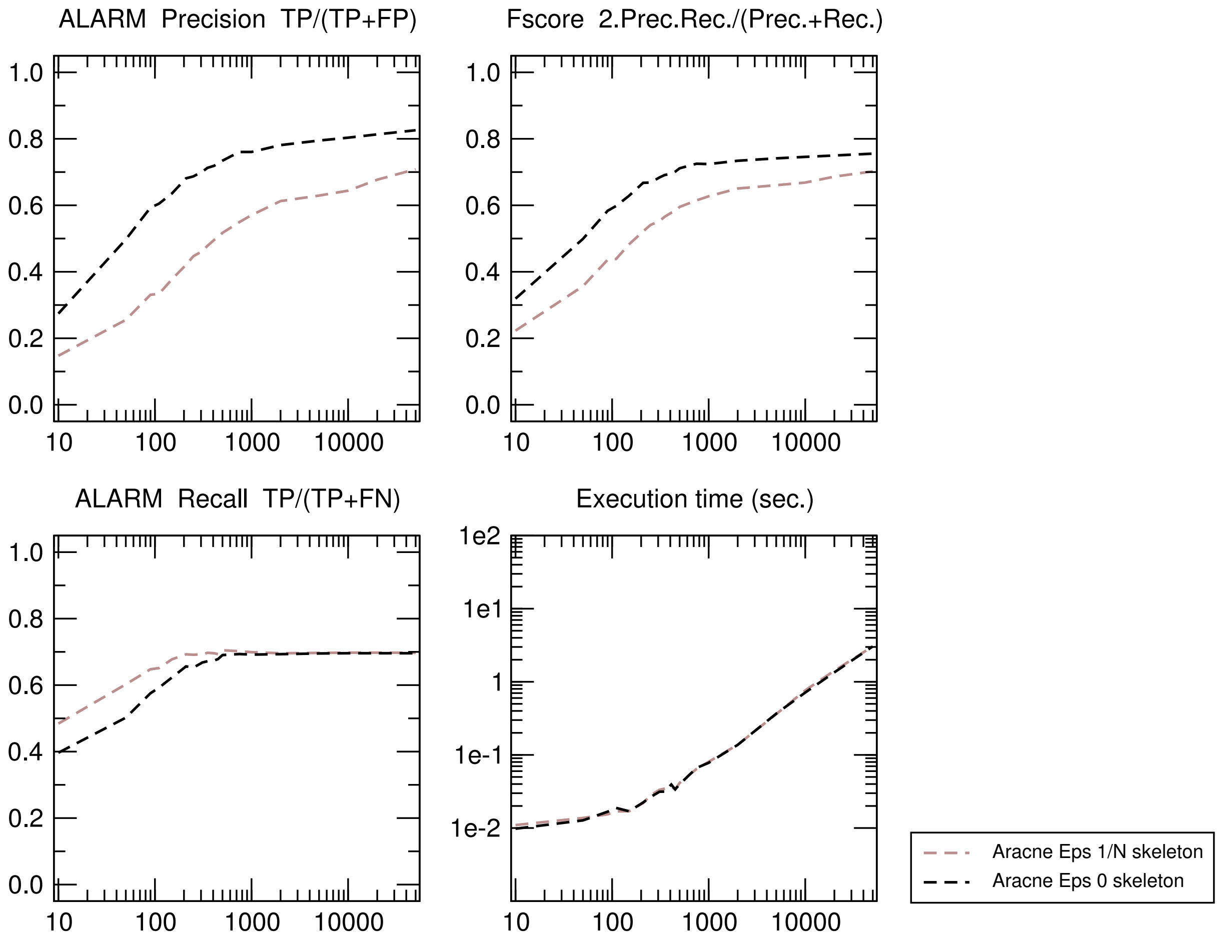

Figure S6: **ALARM network** [37 nodes, 46 links, 509 parameters, Average degree 2.49, Maximum in-degree 4]. Precision, Recall, F-score and execution time for the reconstruction of the skeletons (dashed lines) using the Aracne inference approach.

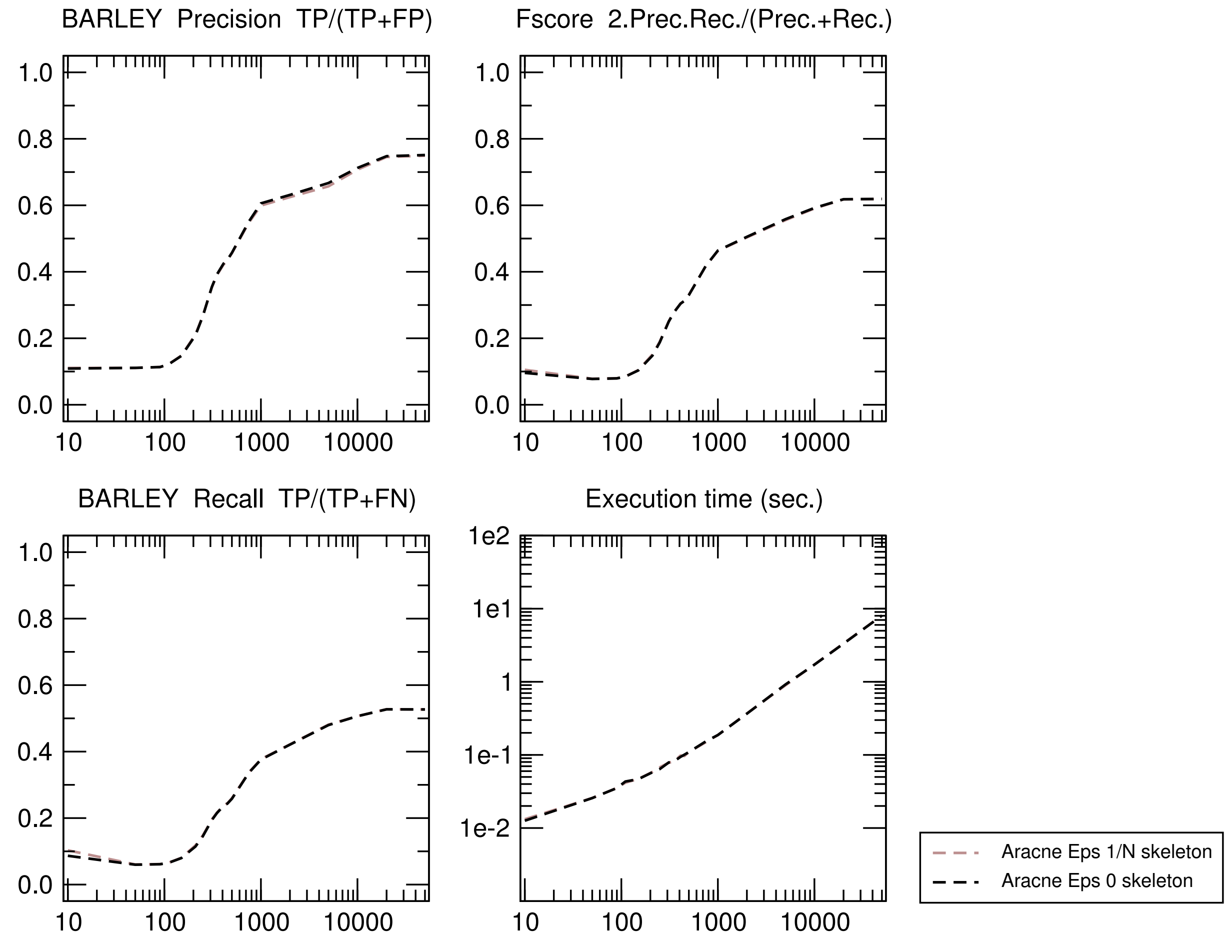

Figure S7: **BARLEY network** [48 nodes, 84 links, 114,005 parameters, Average degree 3.5, Maximum in-degree 4]. Precision, Recall, F-score and execution time for the reconstruction of the skeletons (dashed lines) using the Aracne inference approach.

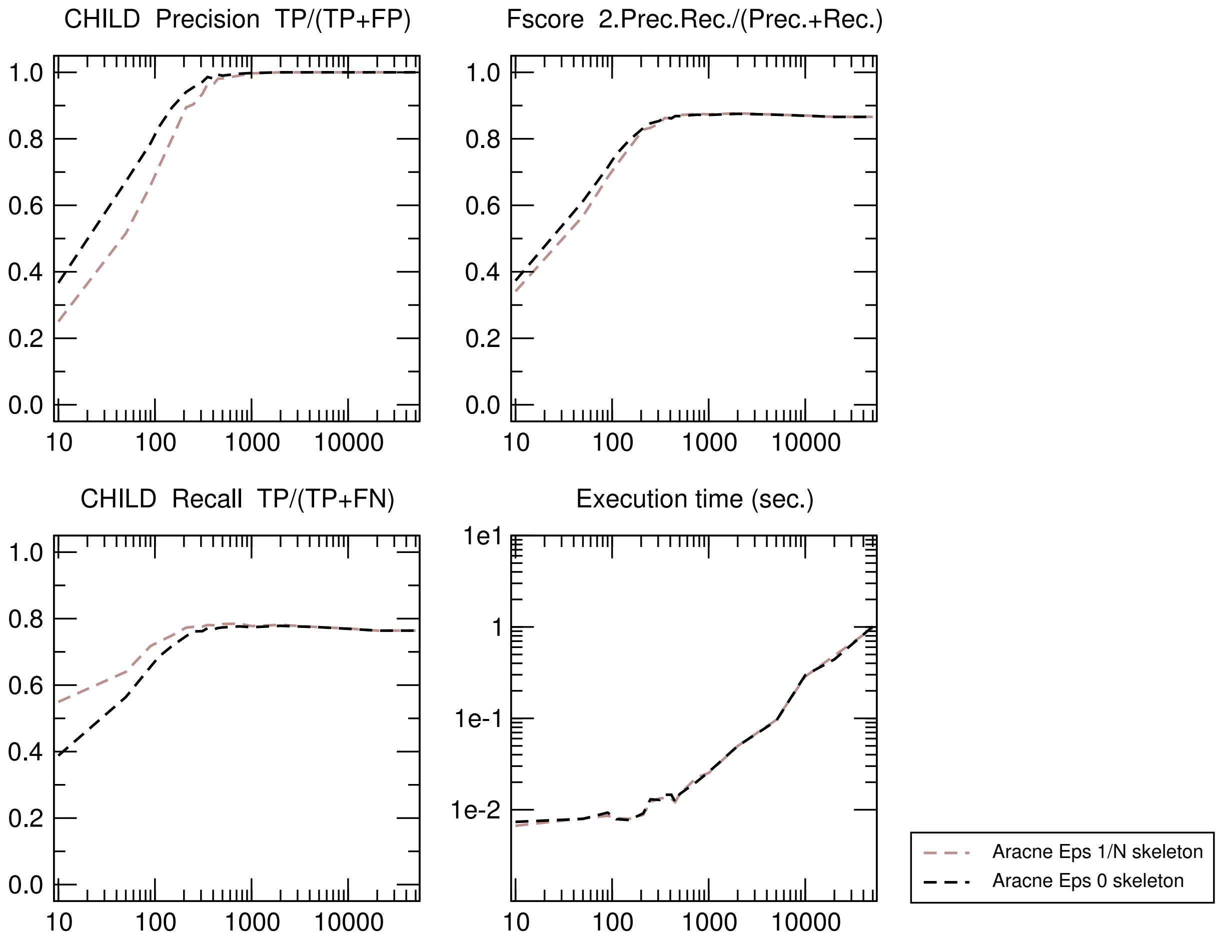

Figure S8: **CHILD network** [20 nodes, 25 links, 230 parameters, Average degree 2.5, Maximum in-degree 2]. Precision, Recall, F-score and execution time for the reconstruction of the skeletons (dashed lines) using the Aracne inference approach.

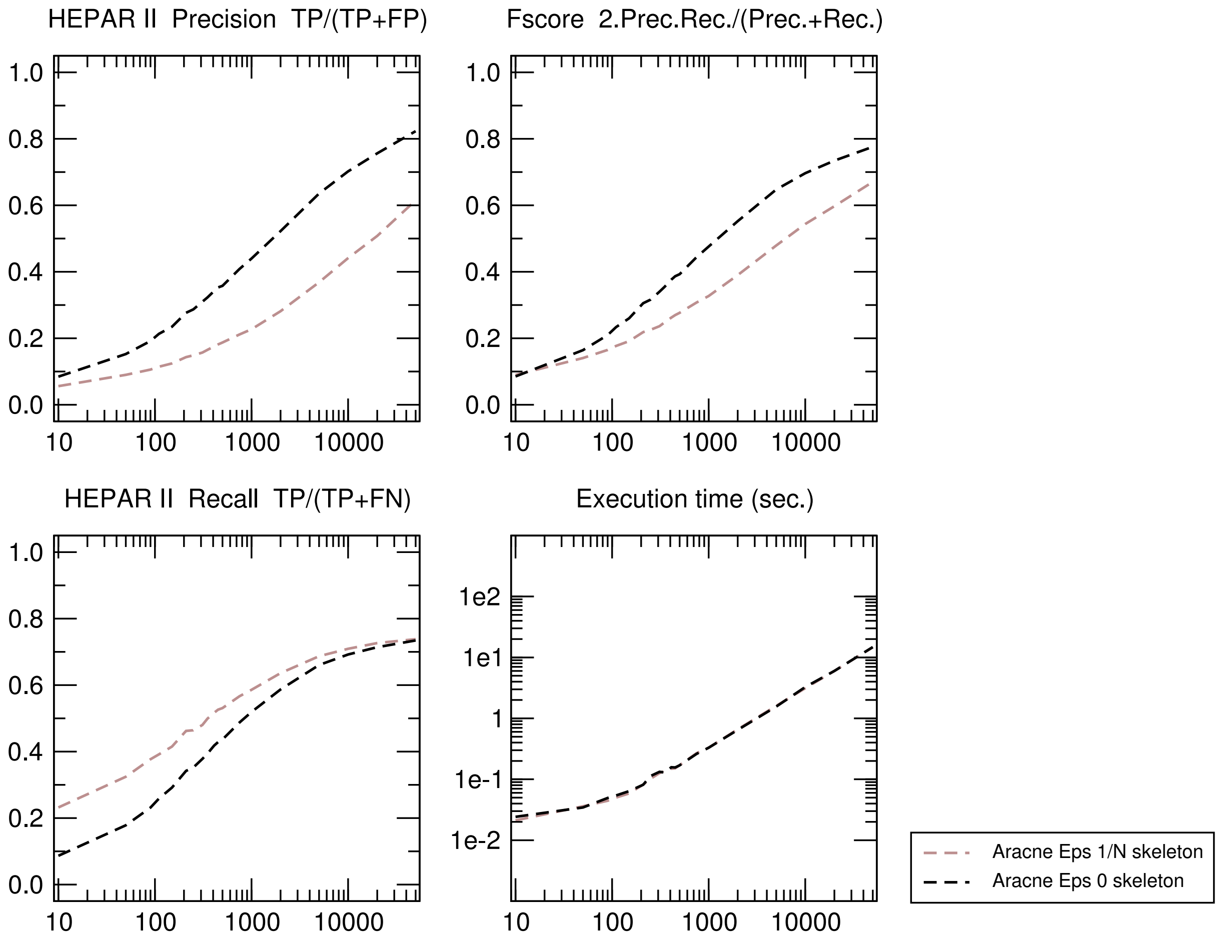

Figure S9: **HEPAR II network** [70 nodes, 123 links, 1,453 parameters, Average degree 3.51, Maximum in-degree 6]. Precision, Recall, F-score and execution time for the reconstruction of the skeletons (dashed lines) using the Aracne inference approach.

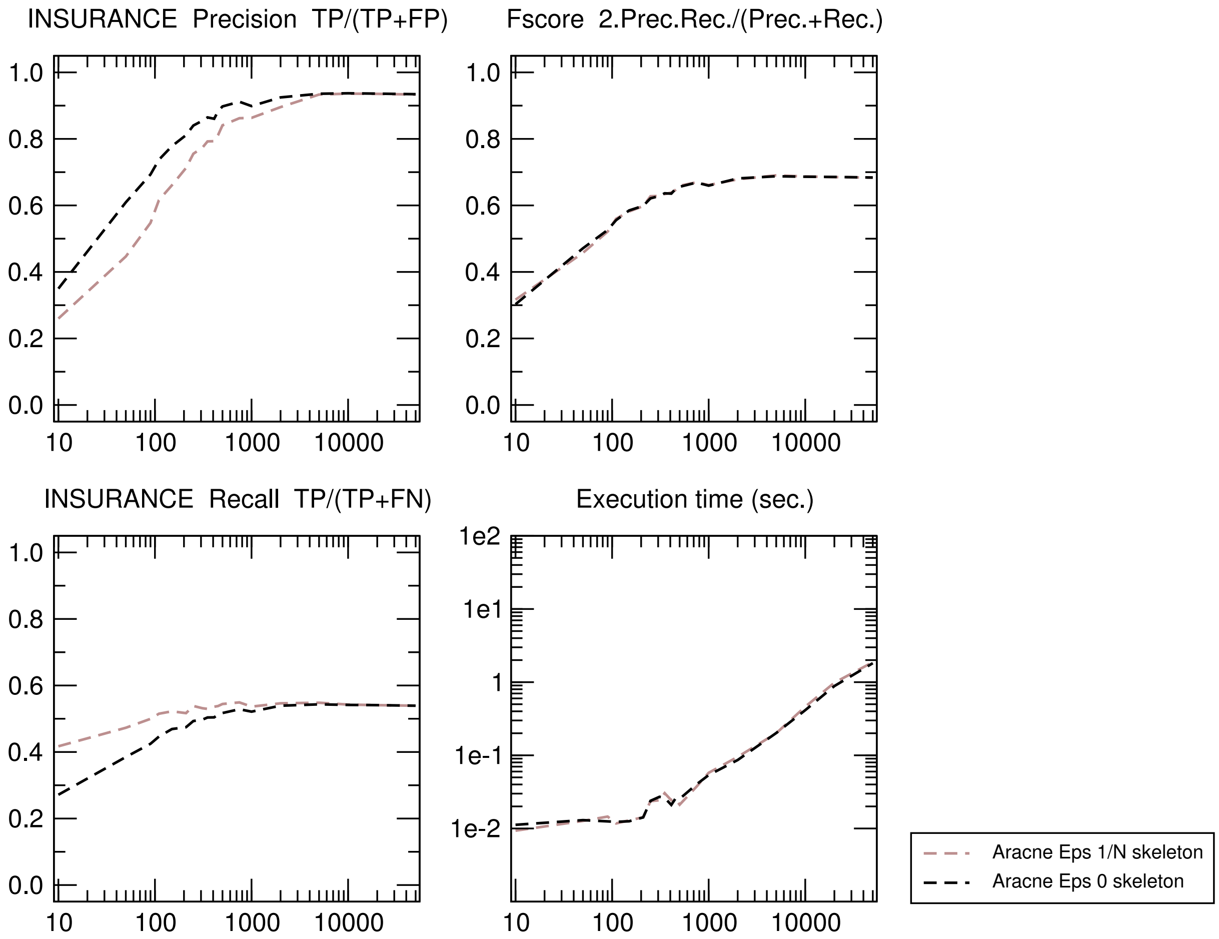

Figure S10: **INSURANCE network** [27 nodes, 52 links, 984 parameters, Average degree 3.85, Maximum in-degree 3]. Precision, Recall, F-score and execution time for the reconstruction of the skeletons (dashed lines) using the Aracne inference approach.

3 Evaluation of the Bayesian methods by score

In this section, the results of the Bayesian inference method using a hill-climbing (HC) heuristics with 100 random restarts [9], as implemented in the `bnlearn` package [5], are evaluated using the following parameter values:

- Bayesian Dirichlet equivalent (BDe) score (**HC BDe**)
- Akaike Information Criteria (AIC) score (**HC AIC**)
- Bayesian Information Criteria (BIC) score (**HC BIC**)

As shown in Figures S11-S15, depending on the underlying causal network, one has to choose the most suitable score for the hill-climbing heuristic approach to output its best reconstruction. The AIC score should be preferred for INSURANCE (Figure S15), the BIC score for the ALARM and HEPAR II (Figures S11 & S14) and the BDe score for the CHILD and BARLEY networks (Figures S13 & S12).

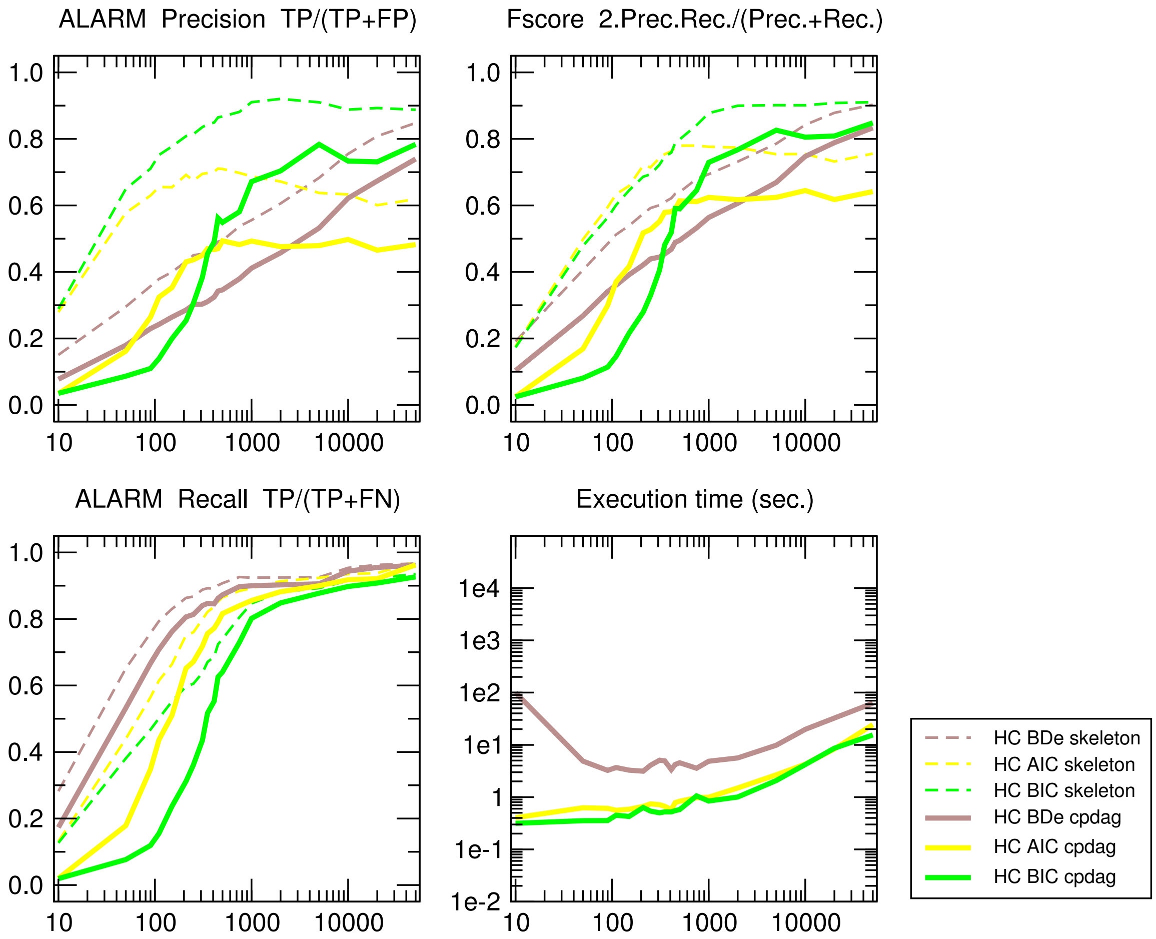

Figure S11: **ALARM network** [37 nodes, 46 links, 509 parameters, Average degree 2.49, Maximum in-degree 4]. Precision, Recall, F-score and execution time for the reconstruction of the skeletons (dashed lines) and CPDAGs (filled lines) using the Bayesian inference approach with BDe, AIC or BIC scores. The Bayesian inference method using the hill climbing heuristics and the BDe score did not converge for 4 datasets out of 50 at sample size $N = 10$. For these non-converging reconstructions, the execution time has been set to $3,600$ seconds.

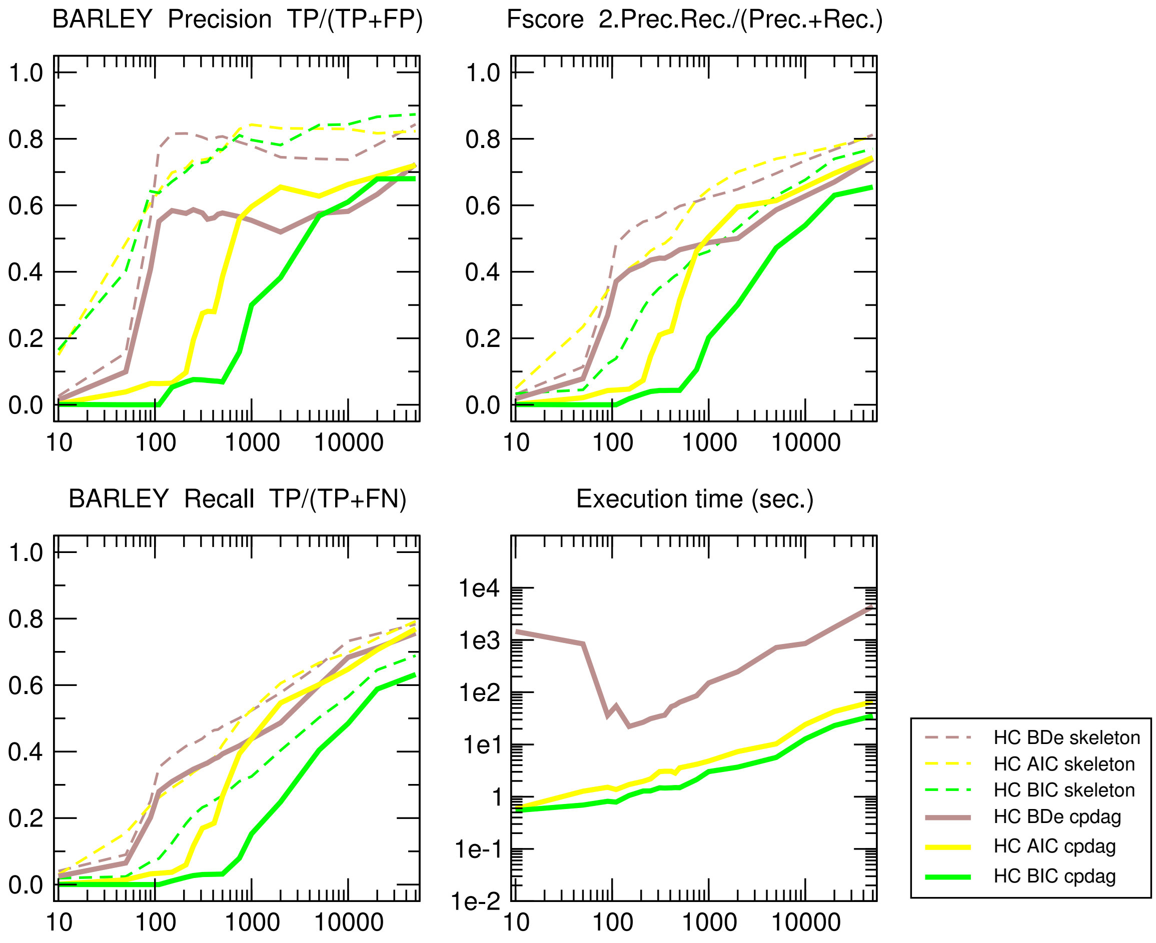

Figure S12: **BARLEY network** [48 nodes, 84 links, 114,005 parameters, Average degree 3.5, Maximum in-degree 4]. Precision, Recall, F-score and execution time for the reconstruction of the skeletons (dashed lines) and CPDAGs (filled lines) using the Bayesian inference approach with BDe, AIC or BIC scores. The Bayesian inference method using the hill climbing heuristics and the BDe score did not converge for (i) 41 datasets out of 50 at sample size $N = 10$, (ii) 36 datasets out of 50 at sample size $N = 50$, (iii) 13 datasets out of 50 at sample size $N = 90$ and (iv) 1 dataset out of 50 at sample size $N = 110$. For these non-converging reconstructions, the execution time has been set to $3,600$ seconds.

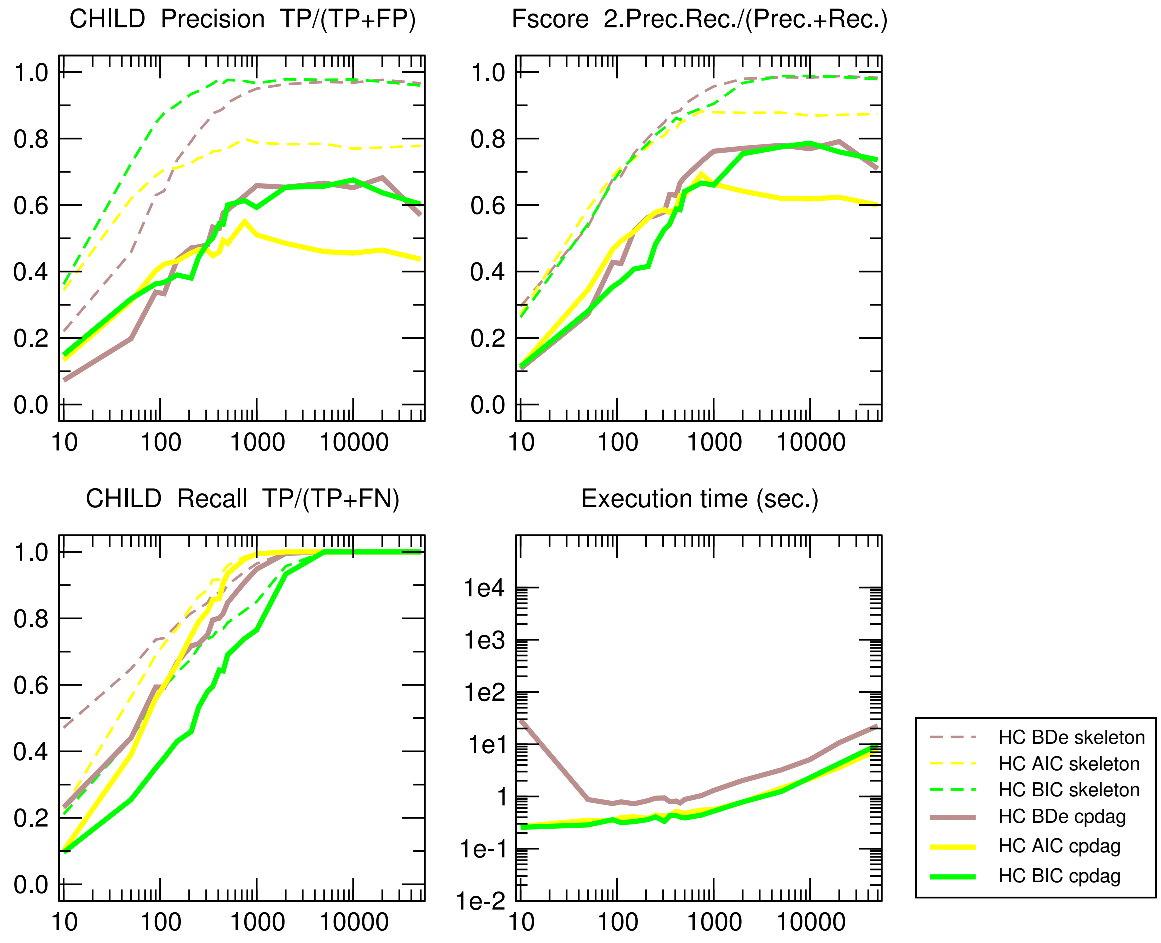

Figure S13: **CHILD network** [20 nodes, 25 links, 230 parameters, Average degree 2.5, Maximum in-degree 2]. Precision, Recall, F-score and execution time for the reconstruction of the skeletons (dashed lines) and CPDAGs (filled lines) using the Bayesian inference approach with BDe, AIC or BIC scores.

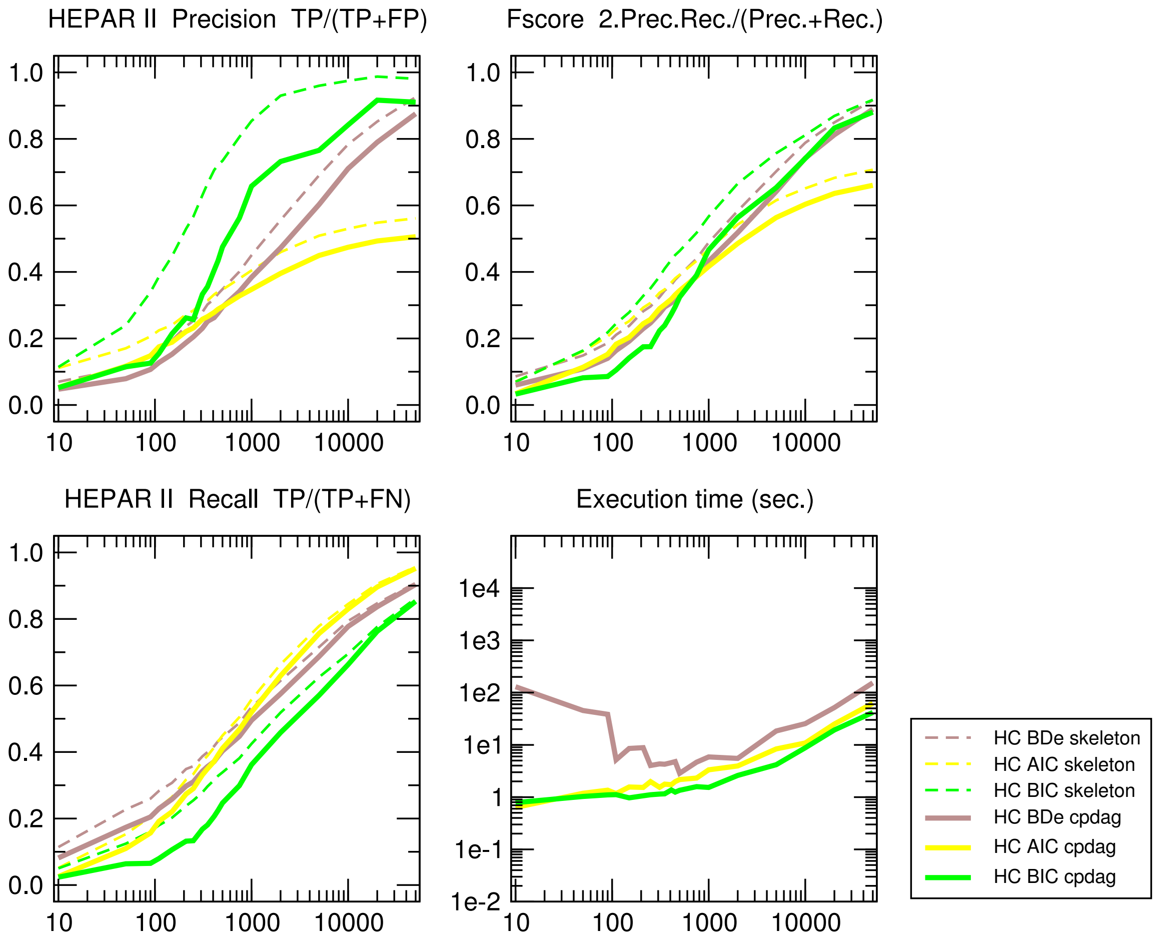

Figure S14: **HEPAR II network** [70 nodes, 123 links, 1,453 parameters, Average degree 3.51, Maximum in-degree 6]. Precision, Recall, F-score and execution time for the reconstruction of the skeletons (dashed lines) and CPDAGs (filled lines) using the Bayesian inference approach with BDe, AIC or BIC scores. The Bayesian inference method using the hill climbing heuristics and the BDe score did not converge for 5 datasets out of 50 at sample size $N = 10$. For these non-converging reconstructions, the execution time has been set to $3,600$ seconds.

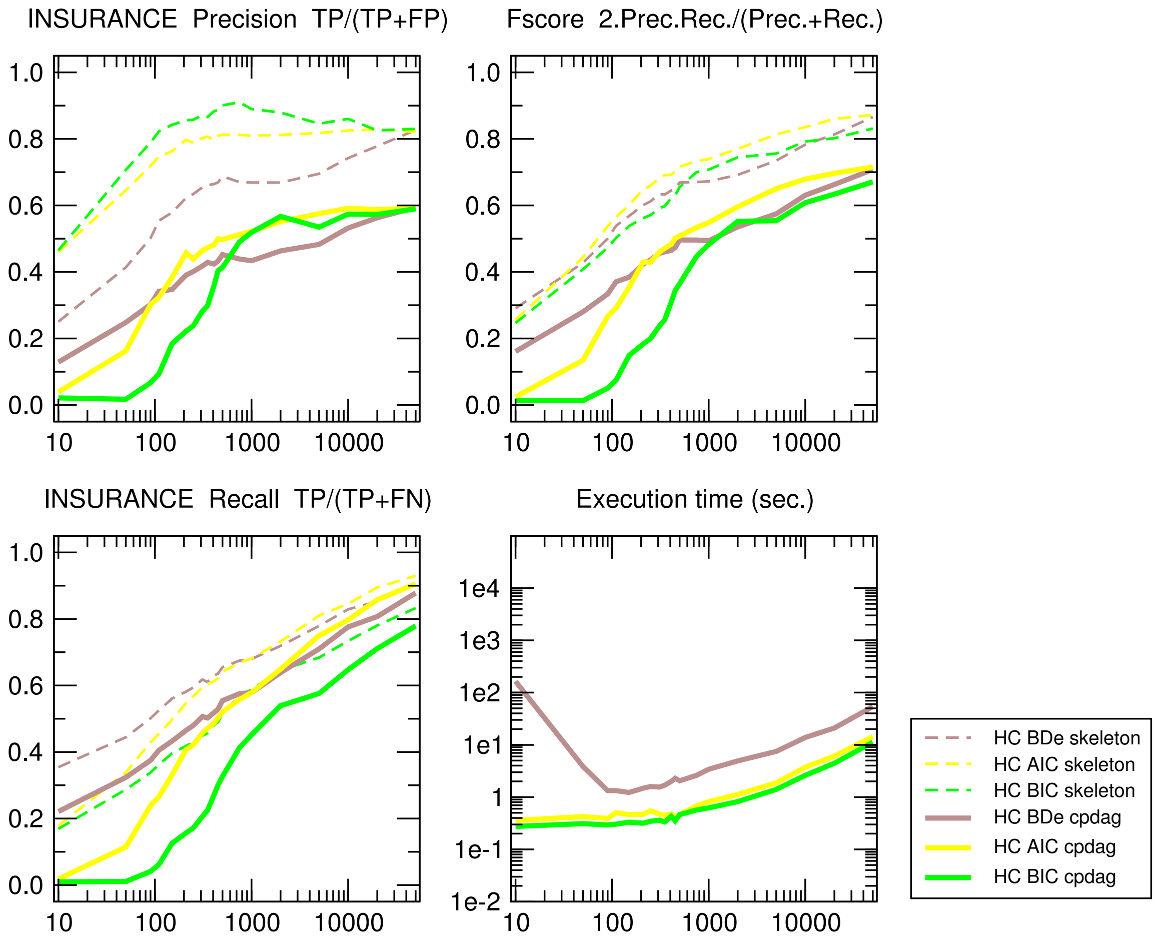

Figure S15: **INSURANCE network** [27 nodes, 52 links, 984 parameters, Average degree 3.85, Maximum in-degree 3]. Precision, Recall, F-score and execution time for the reconstruction of the skeletons (dashed lines) and CPDAGs (filled lines) using the Bayesian inference approach with BDe, AIC or BIC scores.

4 Evaluation of 3off2 by score

In this section, the results of the 3off2 inference approach are evaluated using the following parameter values:

- 3off2 **MDL(rank I)**: 3off2 inference approach using Minimum Description Length (MDL) criteria and *non-shifted* 2-point and 3-point information terms in the rank of individual edges
- 3off2 **MDL(rank I')**: 3off2 inference approach using Minimum Description Length (MDL) criteria and *shifted* 2-point and 3-point information terms in the rank of individual edges
- 3off2 **NML(rank I)**: 3off2 inference approach using Normalized Maximum Likelihood (NML) criteria and *non-shifted* 2-point and 3-point information terms in the rank of individual edges
- 3off2 **NML(rank I')**: 3off2 inference approach using Normalized Maximum Likelihood (NML) criteria and *shifted* 2-point and 3-point information terms in the rank of individual edges

As discussed in the Methods section (see main text), Figures S16-S20 show that the best results on benchmark networks are obtained with the NML score. The MDL score leads to equivalent results, as expected, in the limit of very large datasets (see Appendix). However, with smaller datasets, the most reliable results with the MDL score are obtained using *non-shifted* instead of shifted 2-point and 3-point information terms in the 3off2 rank of individual edges, Eq. 21. This is because the MDL complexity tends to underestimate the importance of edges between nodes with many levels (see Appendix). For finite datasets, it easily leads to spurious conditional independencies, $I'(x;y|\{ui\}) < 0$, when using shifted 2-point and 3-point information, Eq. 22, whereas using non-shifted information in the 3off2 ranks (Eq. 21) tends to limit the number of false negatives as early errors in $\{u_i\}$ can only increase $I(x;y|\{ui\}) \geqslant 0$, in the end, in Eq. 23.

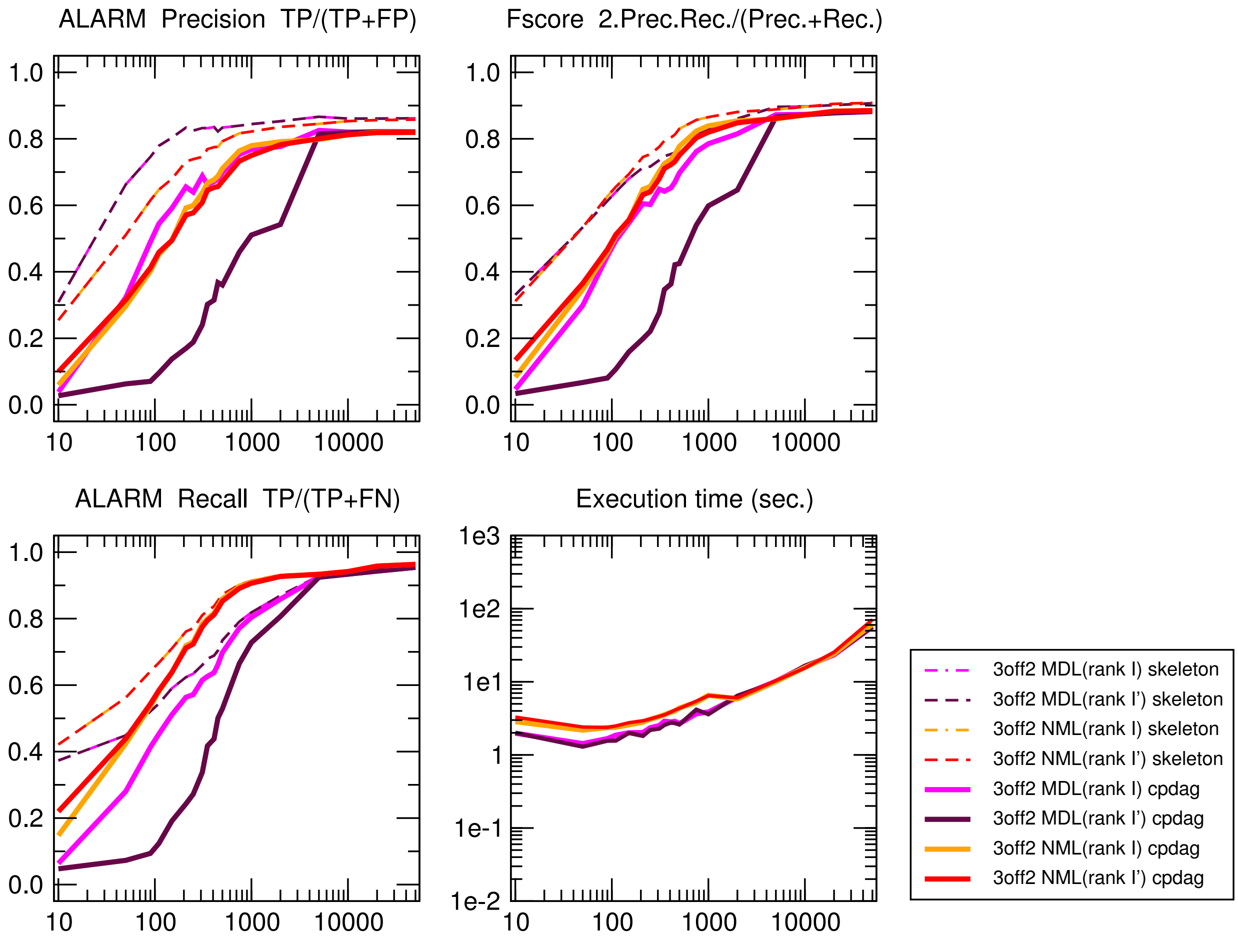

Figure S16: **ALARM network** [37 nodes, 46 links, 509 parameters, Average degree 2.49, Maximum in-degree 4]. Precision, Recall, F-score and execution time for the reconstruction of the skeletons (dashed lines) and CPDAGs (filled lines) using the 3off2 inference approach.

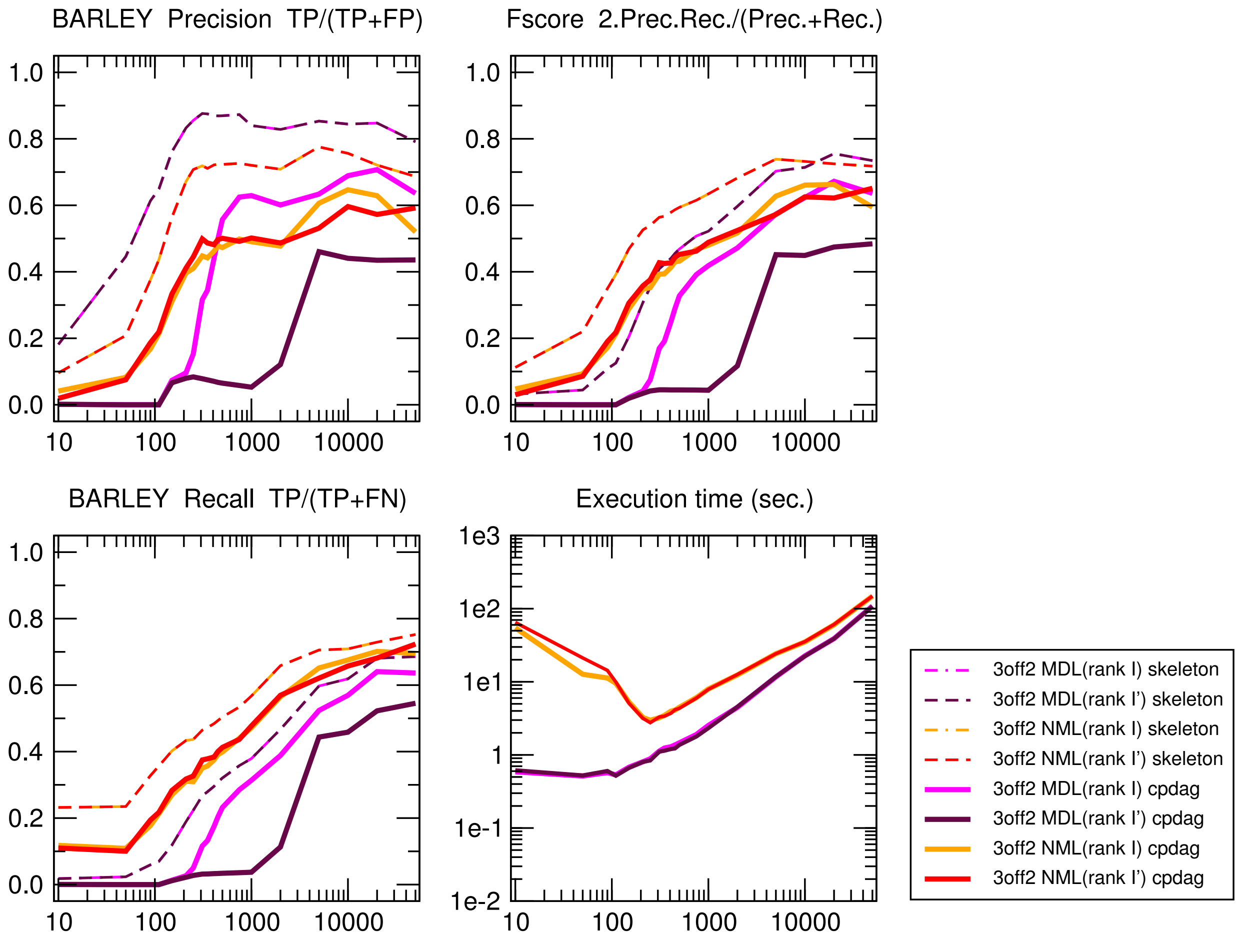

Figure S17: **BARLEY network** [48 nodes, 84 links, 114,005 parameters, Average degree 3.5, Maximum in-degree 4]. Precision, Recall, F-score and execution time for the reconstruction of the skeletons (dashed lines) and CPDAGs (filled lines) using the 3off2 inference approach.

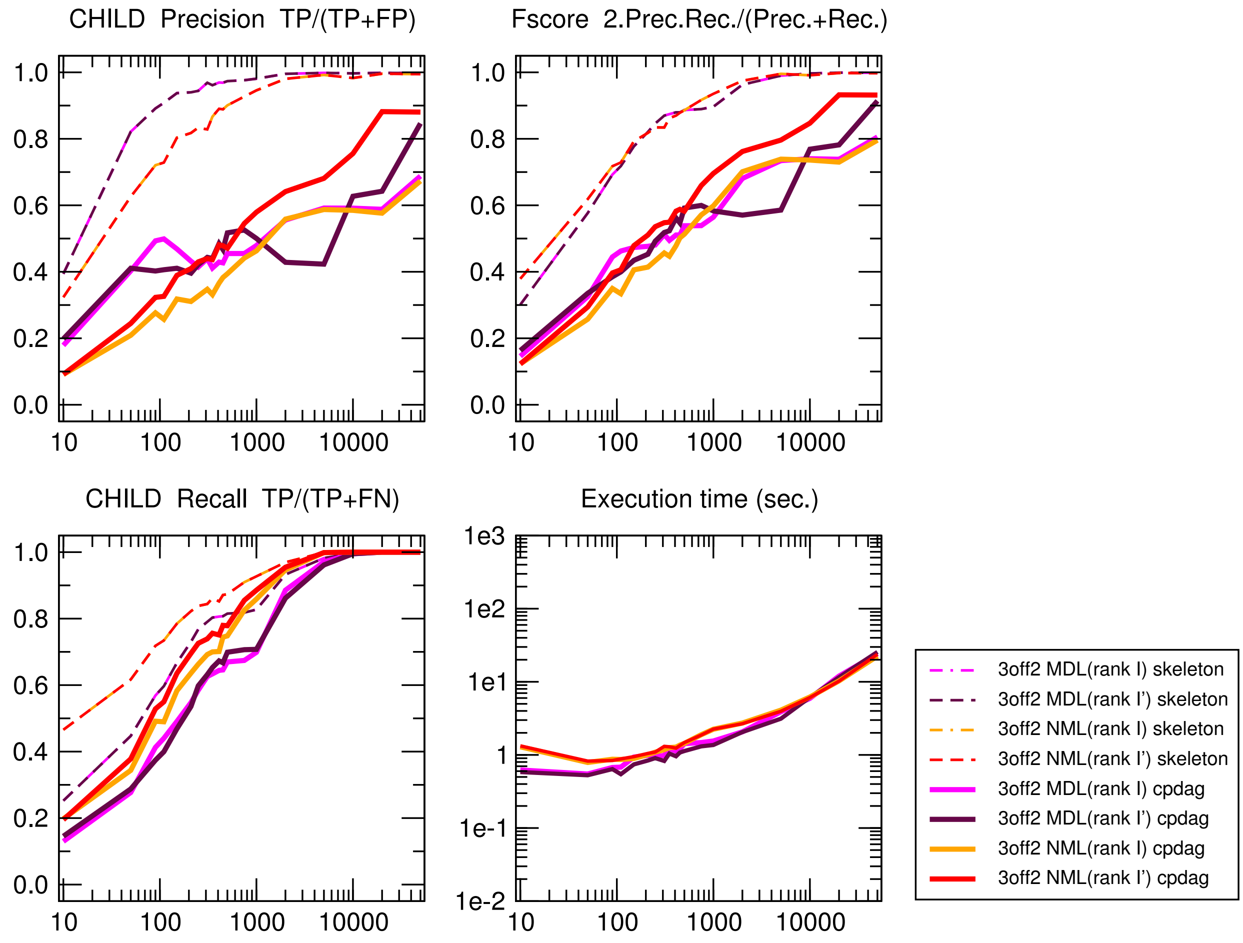

Figure S18: **CHILD network** [20 nodes, 25 links, 230 parameters, Average degree 2.5, Maximum in-degree 2]. Precision, Recall, F-score and execution time for the reconstruction of the skeletons (dashed lines) and CPDAGs (filled lines) using the 3off2 inference approach.

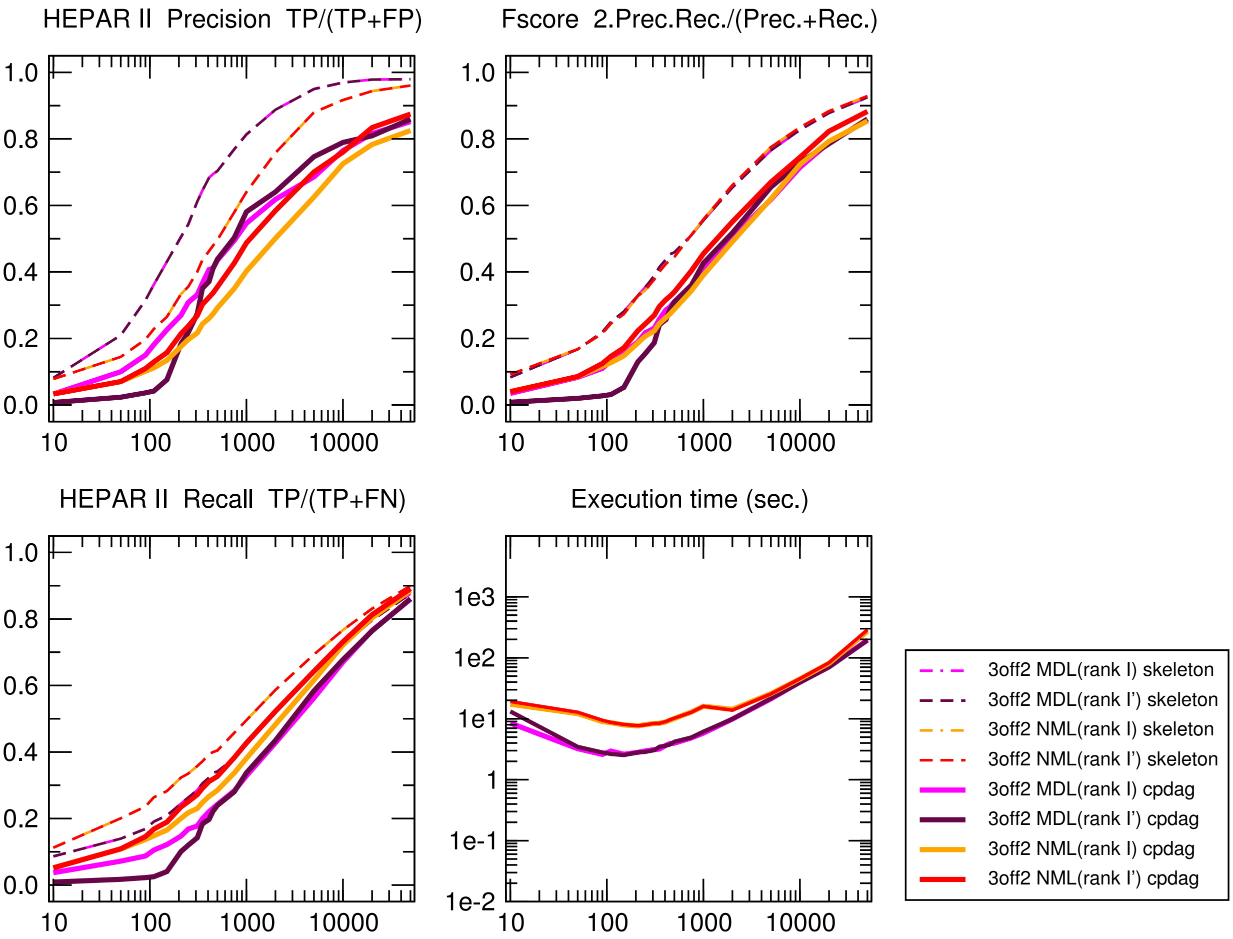

Figure S19: **HEPAR II network** [70 nodes, 123 links, 1,453 parameters, Average degree 3.51, Maximum in-degree 6]. Precision, Recall, F-score and execution time for the reconstruction of the skeletons (dashed lines) and CPDAGs (filled lines) using the 3off2 inference approach.

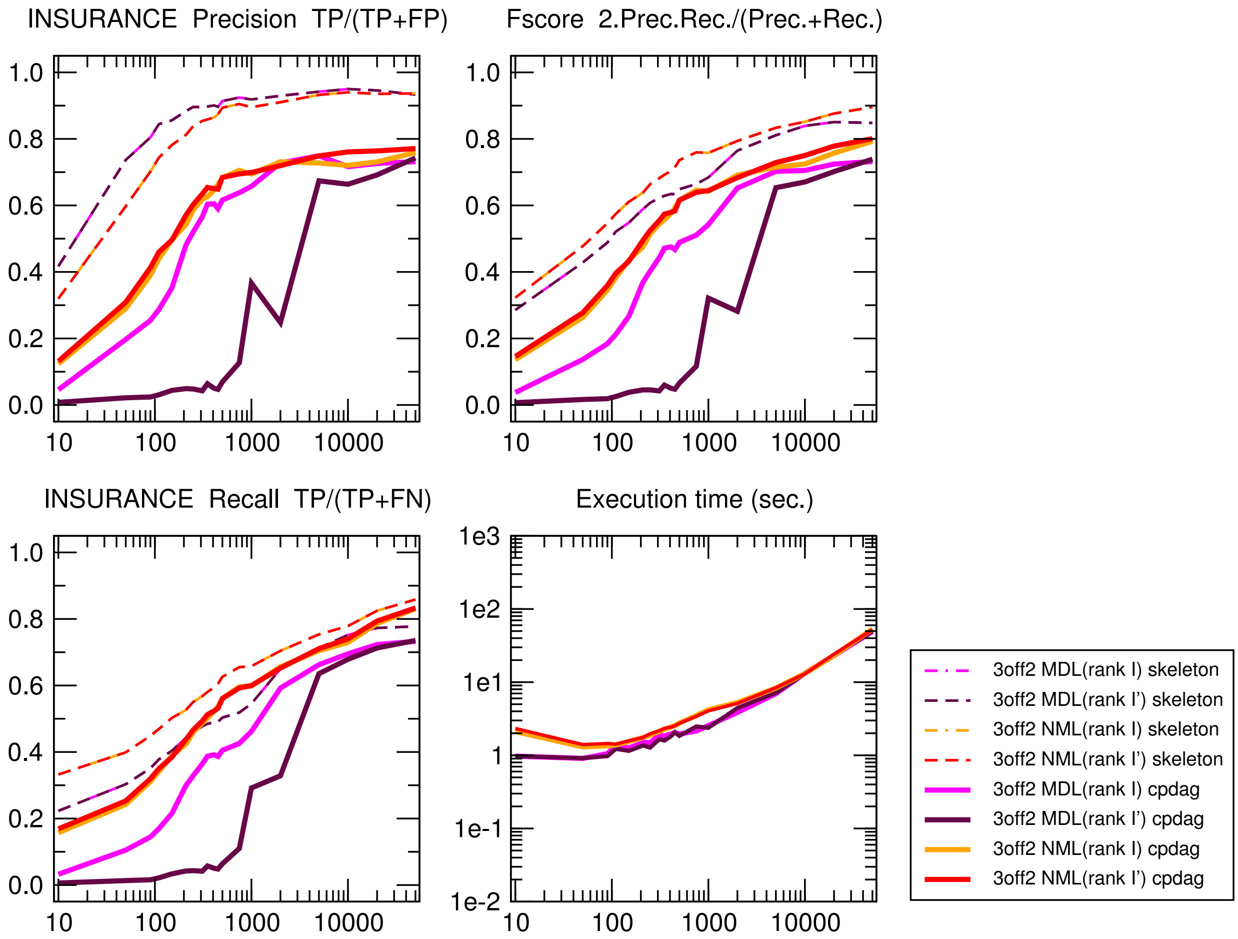

Figure S20: **INSURANCE network** [27 nodes, 52 links, 984 parameters, Average degree 3.85, Maximum in-degree 3]. Precision, Recall, F-score and execution time for the reconstruction of the skeletons (dashed lines) and CPDAGs (filled lines) using the 3off2 inference approach.

5 Evaluation of 3off2 against Bayesian and MMHC methods

In this section, the results of the 3off2 inference approach are evaluated against the Bayesian inference and the MMHC [8] methods using the following parameter values:

- **HC BIC**: Bayesian inference using Bayesian Information Criteria (BIC) score and hill-climbing (HC) heuristics with 100 random restarts [9] as implemented in the `bnlearn` package [5]
- **MMHC BIC**: Hybrid approach [8] combining constraint-based and Bayesian approaches by first identifying both parents and children of each node of the underlying graphical model and then performing a greedy Bayesian hill-climbing search restricted to the identified parents and children of each node. We have used the BIC criteria with significance parameter $\alpha = 0.1$ (using BDe criteria and the range $\alpha = 0.001 - 0.1$ gives very similar results for all tested benchmarks, not shown). The MMHC method is implemented in the `bnlearn` package [5].
- 3off2 **MDL(rank I)**: 3off2 inference approach using Minimum Description Length (MDL) criteria and *non-shifted* 2-point and 3-point information terms in the rank of individual edges

As discussed in Methods section (see main article), the MDL complexity using shifted 2-point and 3-point information terms leads the 3off2 inference approach to cumulate false negative edges with smaller datasets. Yet, the 3off2 algorithm gives good results when using the MDL criteria with *non-shifted* 2-point and 3-point information terms in the rank of individual edges. As shown in Figures S21-S25, CPDAG F-scores of the 3off2 reconstruction method are typically better or comparable to the Bayesian hill-climbing heuristics using the BIC score, except for the CHILD benchmark network, although 3off2 eventually reaches better results than the Bayesian inference approach for very large datasets ($N > 20,000$).

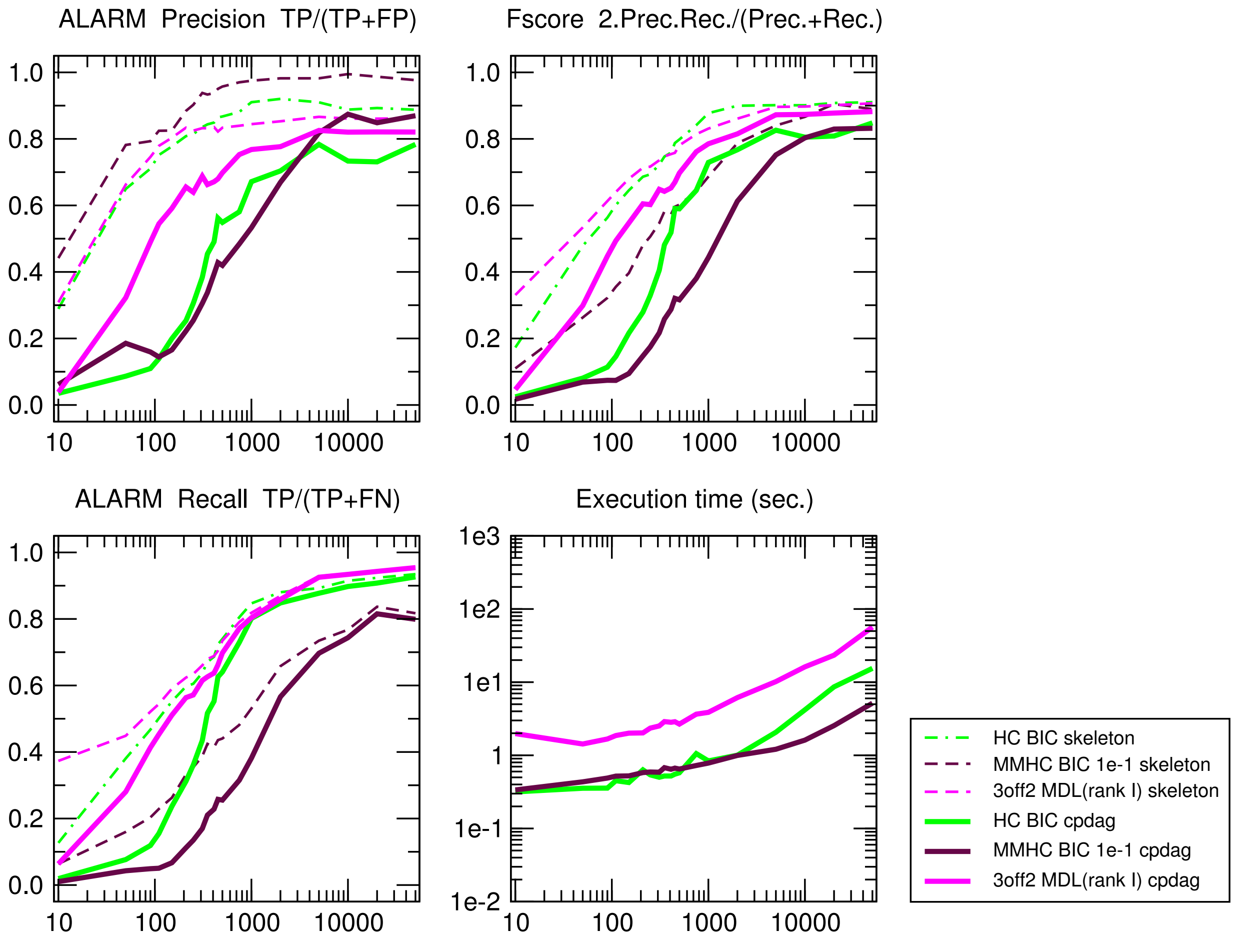

Figure S21: **ALARM network** [37 nodes, 46 links, 509 parameters, Average degree 2.49, Maximum in-degree 4]. Precision, Recall, F-score and execution time for the reconstruction of the skeletons (dashed lines) and CPDAGs (filled lines) using 3off2, Bayesian hill-climbing and Max-Min Hill-Climbing approaches.

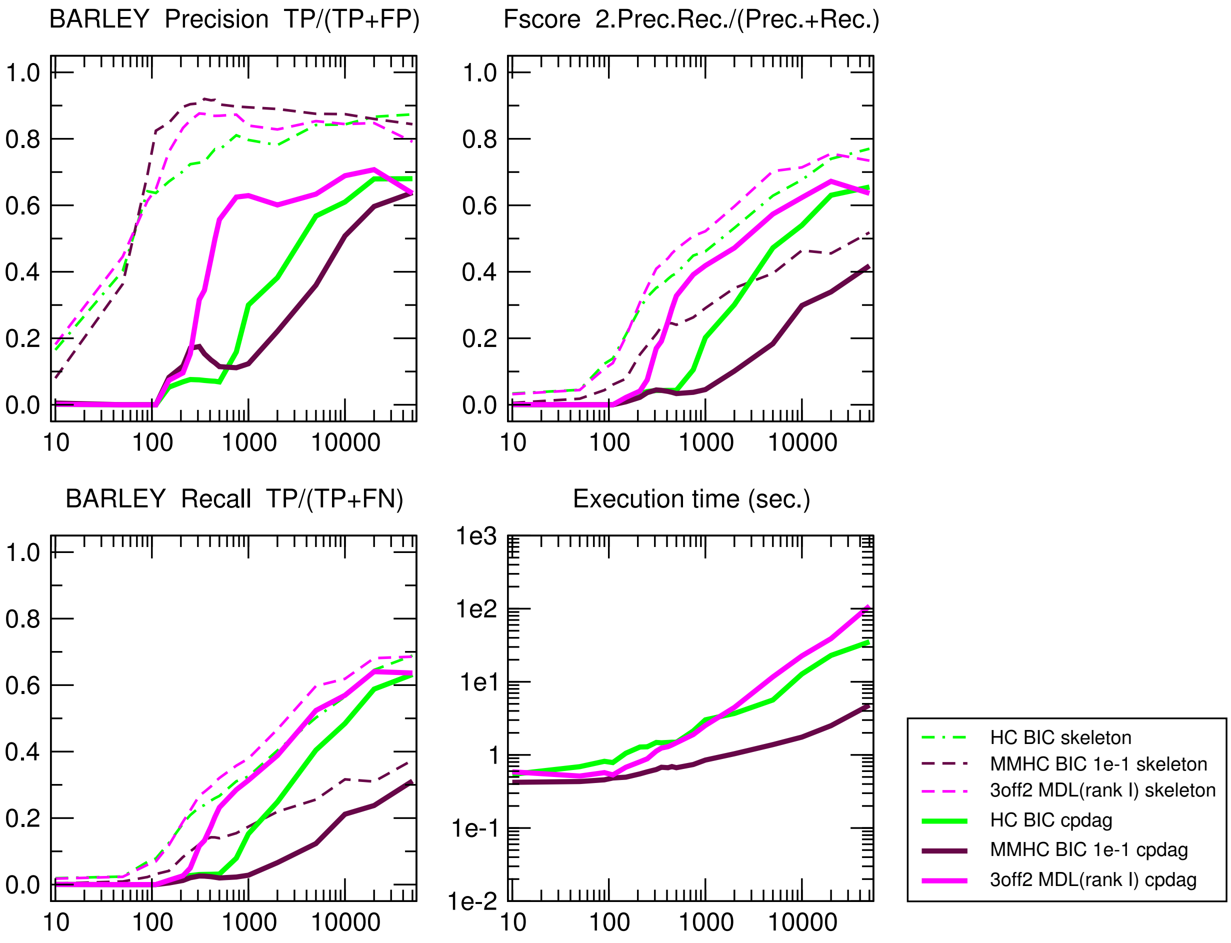

Figure S22: **BARLEY network** [48 nodes, 84 links, 114,005 parameters, Average degree 3.5, Maximum in-degree 4]. Precision, Recall, F-score and execution time for the reconstruction of the skeletons (dashed lines) and CPDAGs (filled lines) using 3off2, Bayesian hill-climbing and Max-Min Hill-Climbing approaches.

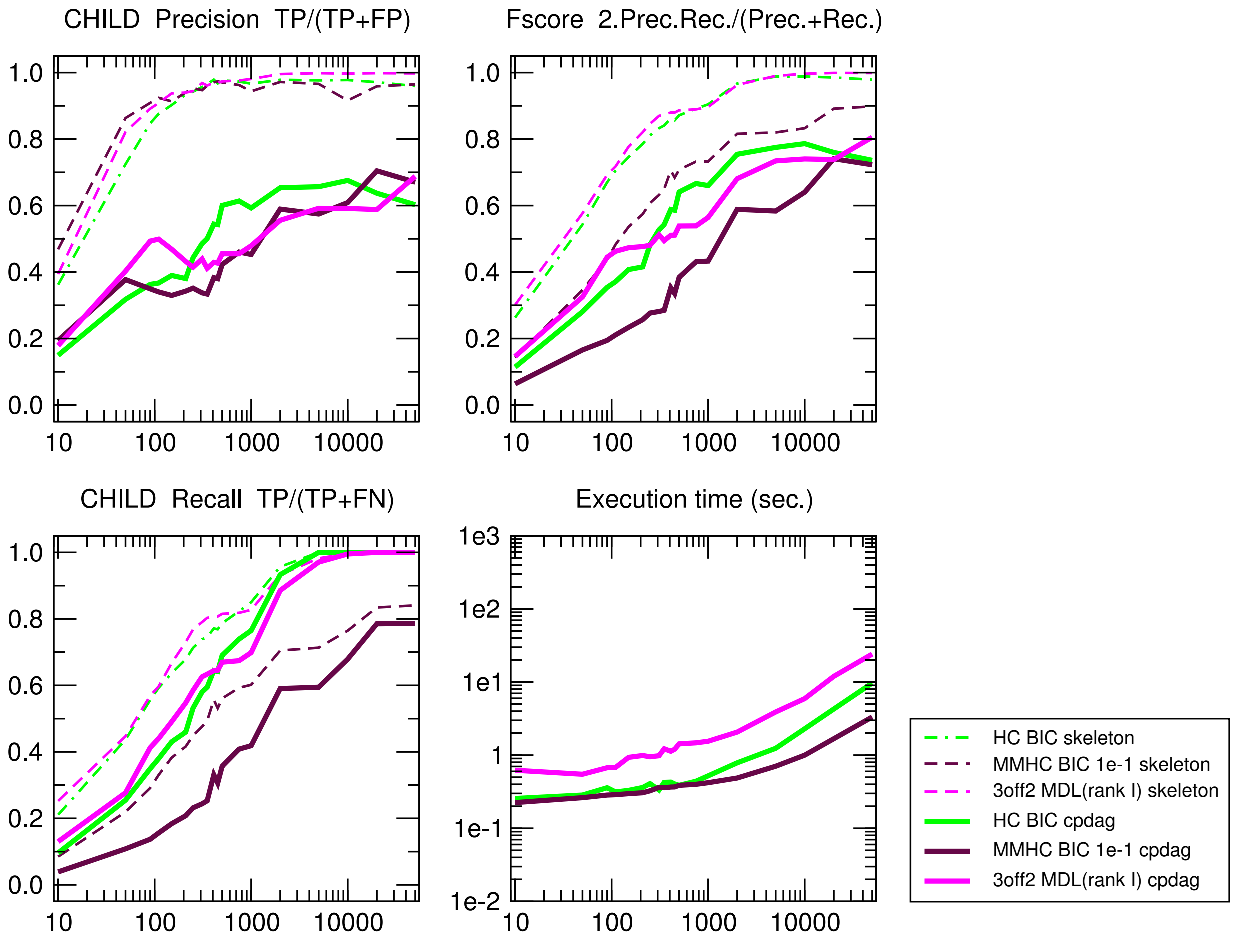

Figure S23: **CHILD network** [20 nodes, 25 links, 230 parameters, Average degree 2.5, Maximum in-degree 2]. Precision, Recall, F-score and execution time for the reconstruction of the skeletons (dashed lines) and CPDAGs (filled lines) using 3off2, Bayesian hill-climbing and Max-Min Hill-Climbing approaches.

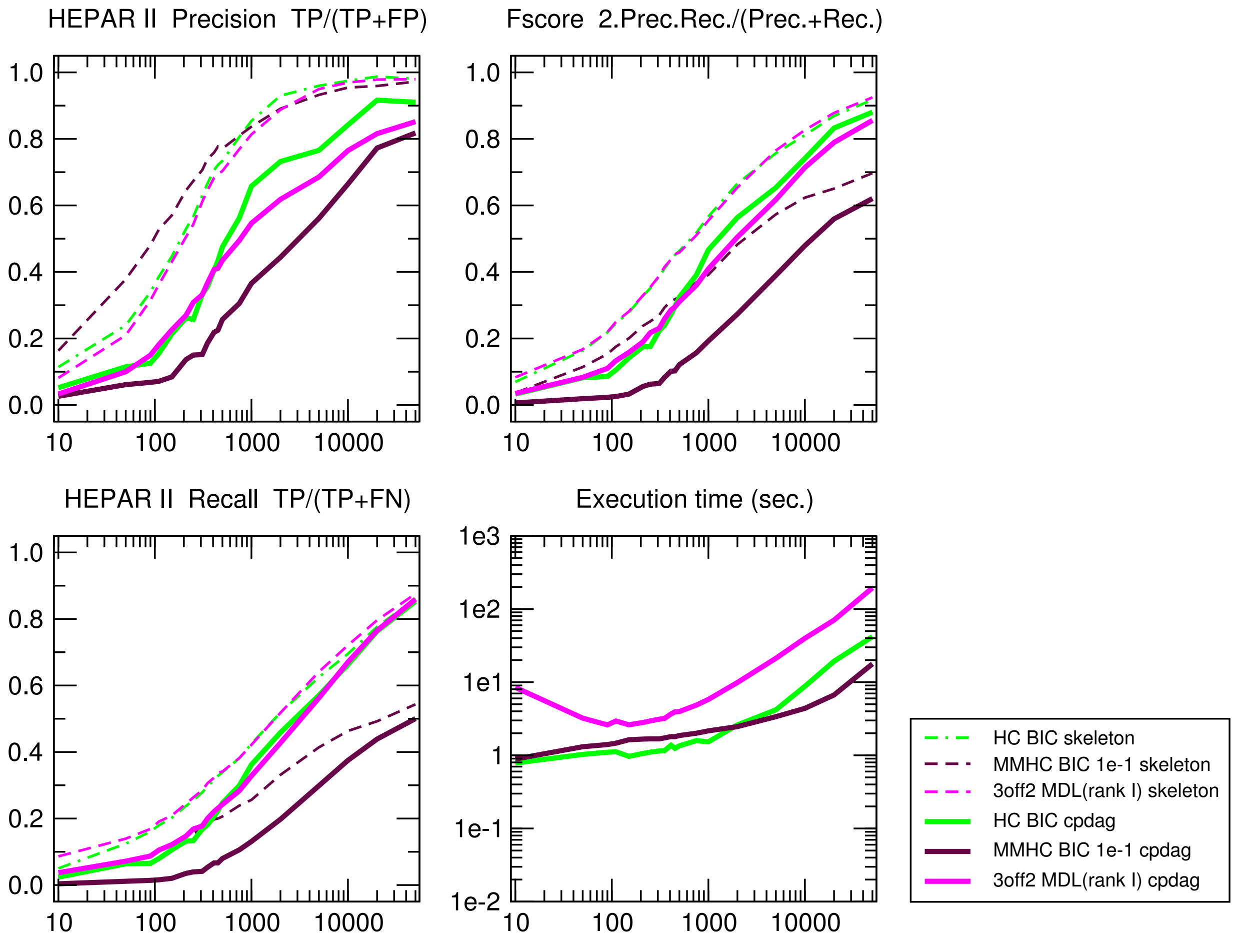

Figure S24: **HEPAR II network** [70 nodes, 123 links, 1,453 parameters, Average degree 3.51, Maximum in-degree 6]. Precision, Recall, F-score and execution time for the reconstruction of the skeletons (dashed lines) and CPDAGs (filled lines) using 3off2, Bayesian hill-climbing and Max-Min Hill-Climbing approaches.

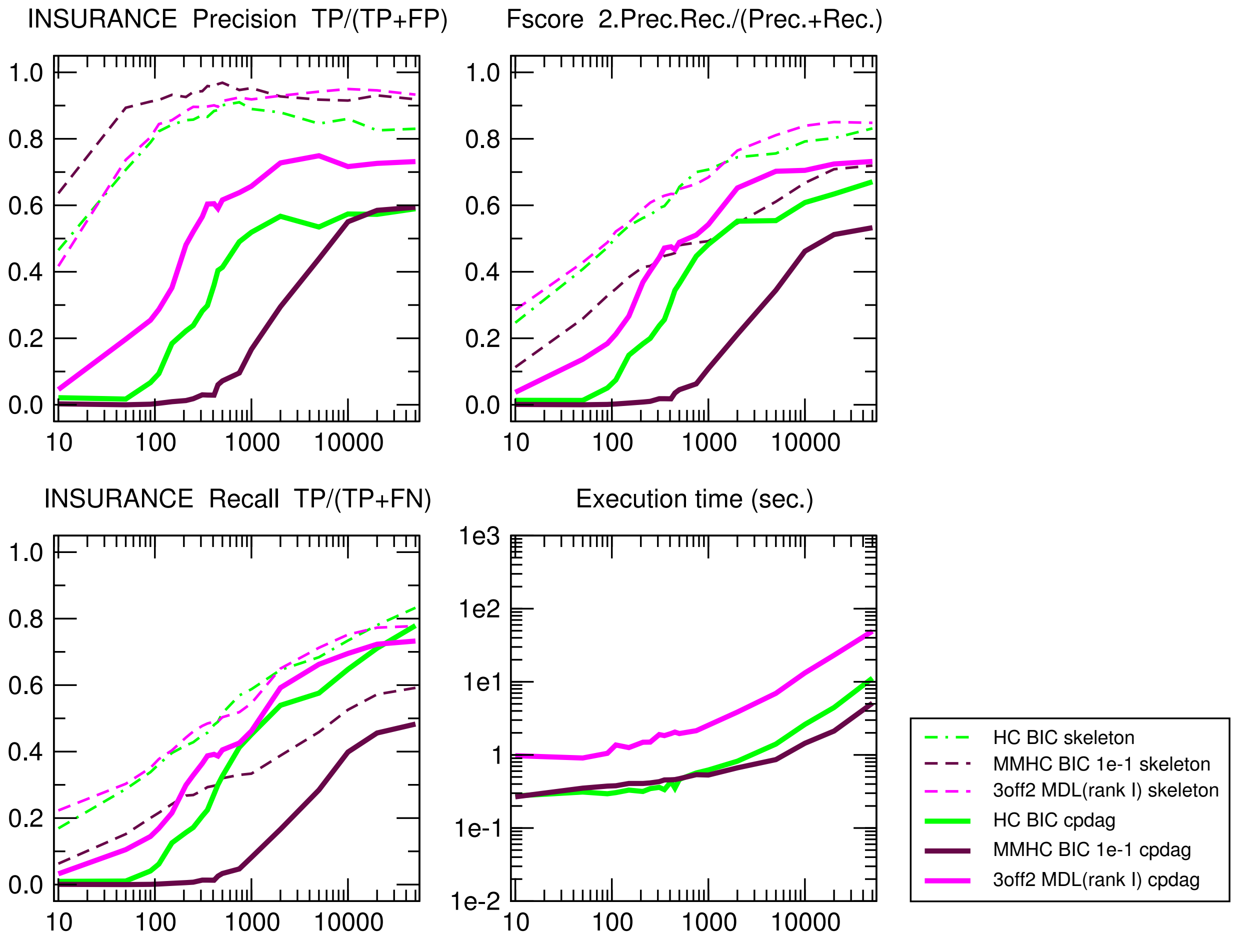

Figure S25: **INSURANCE network** [27 nodes, 52 links, 984 parameters, Average degree 3.85, Maximum in-degree 3]. Precision, Recall, F-score and execution time for the reconstruction of the skeletons (dashed lines) and CPDAGs (filled lines) using 3off2, Bayesian hill-climbing and Max-Min Hill-Climbing approaches.

6 Execution time comparisons

This section compares the execution time of the different inference methods when reconstructing the causal benchmark network used to generate the datasets (or its Markov equivalent graph). The methods and parameter values are:

- 3off2 **MDL(rank I)**: 3off2 inference approach using Minimum Description Length (MDL) criteria and *non-shifted* 2-point and 3-point information terms in the rank of individual edges
- 3off2 **NML(rank I')**: 3off2 inference approach using Normalized Maximum Likelihood (NML) criteria and *shifted* 2-point and 3-point information terms in the rank of individual edges
- **PC 1e-01**: PC inference method [1] implemented in the `pcalg` package [3, 4] with a significance level $\alpha = 0.1$
- **HC BIC**: Bayesian inference using Bayesian Information Criteria (BIC) score and hill-climbing (HC) heuristics with 100 random restarts [9] implemented in the `bnlearn` package [5]
- **MMHC**: Hybrid approach [8] combining constraint-based and Bayesian approaches by first identifying both parents and children of each node of the underlying graphical model and then performing a greedy Bayesian hill-climbing search restricted to the identified parents and children of each node. We have used the BIC criteria with significance parameter $\alpha = 0.1$ (using BDe criteria and the range $\alpha = 0.001 - 0.1$ gives very similar results for all tested benchmarks, not shown). The MMHC method is implemented in the `bnlearn` package [5].

As shown in Figure S26, the execution times for the 3off2 reconstruction method follow typically similar trends (although shifted by a roughly constant factor, $\times$5-10) as Bayesian hill-climbing heuristics or the fast MMHC hybrid method. By contrast, the PC algorithm, which is quite fast for small datasets, becomes significantly slower for larger datasets.

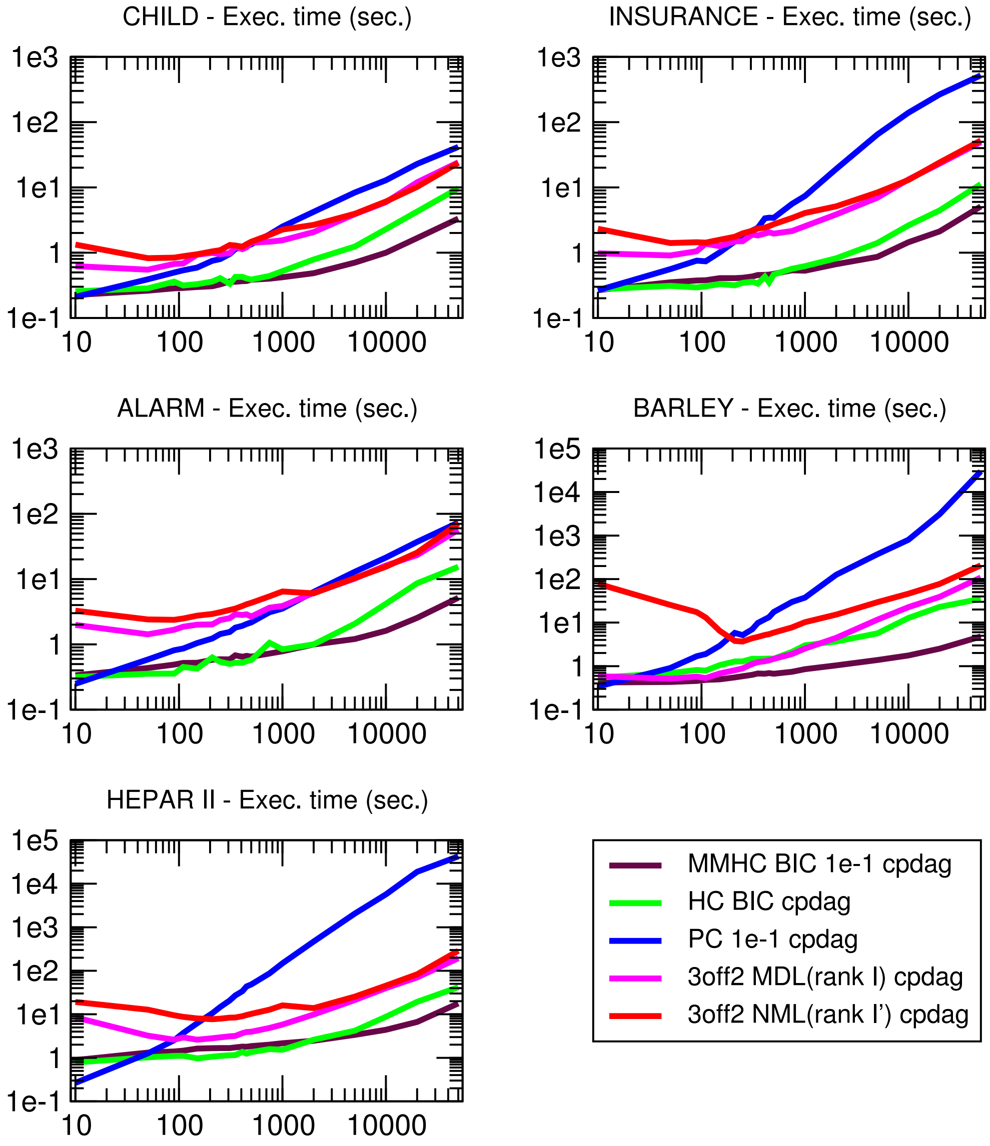

Figure S26: **Network Reconstruction Execution time.** Execution time in seconds for the reconstruction of the five studied benchmark networks.

Statistical Measure	3off2 *NML*	PC $\alpha = 10^{-1}$	PC $\alpha = 10^{-2}$	MMHC *BDe*	MMHC *BIC*	Bayes hc *BDe*	Bayes hc *BIC*	Aracne $\epsilon = 0$
Recall^u	1	0.82	0.64	0.55	0.55	0.91	0.73	0.73
Precision^u	0.65	0.64	0.7	0.55	0.55	0.56	0.67	0.89
Fscore^u	0.79	0.72	0.67	0.55	0.55	0.69	0.70	0.80
Distance^u	0.13	0.16	0.16	0.22	0.22	0.18	0.16	0.11
Recall^d	1	0.6	0.2	0.5	0.44	0.89	0.57	NA
Precision^d	0.41	0.21	0.1	0.45	0.36	0.47	0.33	NA
Fscore^d	0.58	0.31	0.13	0.47	0.4	0.62	0.42	NA
Distance^d	0.22	0.29	0.29	0.24	0.27	0.22	0.24	NA

Table S1: **Interactions reconstructed by 3off2 and four alternative methods on the subnetwork of 11 known regulatory interactions in hematopoiesis (Figure 7).** These calculations were made assuming that the 11 experimentally proven interactions correspond to the true regulatory network (in practice, we expect that some of the inferred edges counted as false positives might in fact turn out to be correctly predicted links). ^{u} indicates that the calculations were made on the undirected network, whereas ^{d} means directed network. The "Distance" measure corresponds to the number of "errors" (including orientations for the directed network comparison) over the total number of possible edges, *i.e.* Distance=$2(FP + FN)/n(n-1)$, where $n = 10$ is the number of nodes in the subnetwork including the 11 known regulatory interactions (Figure 7).

References

[1] Spirtes, P., Glymour, C.: An algorithm for fast recovery of sparse causal graphs. Social Science Computer Review **9**, 62–72 (1991)

[2] Colombo, D., Maathuis, M.H.: Order-independent constraint-based causal structure learning. Journal of Machine Learning Research **15**, 3741–3782 (2014)

[3] Kalisch, M., Mächler, M., Colombo, D., Maathuis, M.H., Bühlmann, P.: Causal inference using graphical models with the r package pcalg. Journal of Statistical Software **47**(11), 1–26 (2012)

[4] Kalisch, M., Bühlmann, P.: Robustification of the pc-algorithm for directed acyclic graphs. Journal Of Computational And Graphical Statistics **17**(4), 773–789 (2008)

[5] Scutari, M.: Learning Bayesian Networks with the bnlearn R Package. Journal of Statistical Software **35**(3), 1–22 (2010)

[6] Margolin, A.A., Nemenman, I., Basso, K., Wiggins, C., Stolovitzky, G., Favera, R., Califano, A.: ARACNE: An Algorithm for the Reconstruction of Gene Regulatory Networks in a Mammalian Cellular Context. BMC Bioinformatics **7**(Suppl 1), 7 (2006)

[7] Meyer, P.E., Lafitte, F., Bontempi, G.: minet: A R/Bioconductor package for inferring large transcriptional networks using mutual information. BMC Bioinformatics **9**, 461 (2008)

[8] Tsamardinos, I., Brown, L.E., Aliferis, C.F.: The Max-Min Hill-Climbing Bayesian Network Structure Learning Algorithm. Machine Learning **65**(1), 31–78 (2006)

[9] Chickering, D.M., Geiger, D., Heckerman, D.: Learning Bayesian networks: Search methods and experimental results. In: Proceedings of the Fifth International Workshop on Artificial Intelligence and Statistics, pp. 112–128 (1995)
